# Self-Efficacy and Performance in Artistic Gymnastics: A Systematic Review

**DOI:** 10.3390/sports14080343

**Published:** 2026-08-07

**Authors:** Federica Marzoli, Gianluca di Pinto, Matteo Campanella, Selenia di Fronso, Maurizio Bertollo, Davide Curzi

**Affiliations:** 1 Department of Economic, Psychological, Communication, Education and Movement Science, University Niccolò Cusano, 00166 Rome, Italy; federica.marzoli@unicusano.it (F.M.); gianluca.dipinto@phd.unich.it (G.d.P.); davide.curzi@unicusano.it (D.C.); 2 Department of Medicine and Aging Sciences, University “G. d’Annunzio” Chieti–Pescara, 66100 Chieti, Italy; maurizio.bertollo@unich.it; 3 Department of Theoretical and Applied Sciences, eCampus University, 22060 Novedrate, Italy; selenia.difronso@uniecampus.it

**Keywords:** self-efficacy, artistic gymnastics, sport performance, task specificity, self-confidence, psychological readiness, systematic review

## Abstract

Self-efficacy is relevant to sport performance, but its role in artistic gymnastics is unclear because studies vary in construct definition, timing, and outcomes. This PRISMA 2020- and PERSIST 2021-informed systematic review was prospectively registered in PROSPERO (CRD420251266838). PubMed, Scopus, Web of Science, and EBSCOhost (SPORTDiscus and APA PsycInfo) were searched from inception to 18 December 2025, supplemented by backward reference-list checking and forward citation searching. Eligible quantitative studies assessed self-efficacy or task- or apparatus-specific efficacy expectations, or used performance predictions or an adapted gymnastics-task confidence measure as efficacy-related proxies and reported a performance outcome; qualitative evidence was contextual only. Nine studies were included (eight quantitative; one qualitative). Competitive studies using direct task- or event-specific efficacy measures or efficacy-related proxies and judged outcomes generally reported positive associations, but correlations and predictive patterns varied across apparatus, samples, and measures. Educational findings and broader confidence constructs were treated as indirect evidence. Meta-analysis was inappropriate because constructs, samples, apparatus, timing, outcomes, and reporting were heterogeneous. The evidence therefore indicates a possible positive but inconsistent, low-certainty association between task- or apparatus-specific efficacy-related beliefs and performance. Contemporary longitudinal studies and standardized gymnastics-specific measures are needed.

## 1. Introduction

Artistic gymnastics is a highly demanding sport characterized by complex motor skills, precise technical execution, and strict evaluative criteria [1]. Performance is judged by experts according to predefined codes, leaving little tolerance for execution errors and requiring athletes to maintain high levels of control, consistency, and concentration throughout routines [2]. Consequently, gymnasts are routinely exposed to psychological pressure, particularly in competitive settings in which performance outcomes are closely scrutinized and publicly evaluated [3]. These demands may be especially salient on apparatus such as balance beam and uneven bars, where technical complexity, postural instability, and the consequences of attentional lapses can make performance execution particularly vulnerable to psychological fluctuations [4,5].

In sport, self-efficacy, self-confidence, competitive anxiety, and emotion regulation have each been associated with performance-related outcomes, although the strength and direction of these relationships vary across contexts, competitive levels, and measurement approaches [6,7,8,9,10,11,12]. In artistic gymnastics, these factors are particularly relevant because routines are pre-planned, sequential, and evaluated according to strict technical criteria. However, the extent to which specific psychological constructs contribute to gymnastics performance remains difficult to establish, partly because studies have used heterogeneous definitions, instruments, timing of assessment, and performance outcomes.

Within this context, self-efficacy represents a theoretically relevant psychological construct. According to Bandura’s social cognitive theory, self-efficacy refers to individuals’ beliefs in their capability to organize and execute the actions required to manage prospective situations [13]. Self-efficacy is inherently task- and context-specific and influences how individuals approach challenges, regulate effort, cope with adversity, and persist in the face of difficulty [14]. In sport psychology, self-efficacy should be distinguished from broader constructs such as sport confidence and state sport confidence [10,12,15], CSAI-based self-confidence [9], and perceived physical ability (Supplementary Table S2), which are not necessarily tied to a specific task, apparatus, or performance criterion. Examples relevant to this review included a balance beam confidence measure adapted from a state sport confidence inventory and treated as an efficacy-related proxy [4], CSAI-based self-confidence [9], broader sport confidence/self-confidence syntheses [10,12], general physical self-efficacy [16], and perceived-ability records documented in Supplementary Table S2. These constructs were not treated as interchangeable with direct task- or apparatus-specific self-efficacy measures; the adapted task-specific confidence measures and predicted scores were classified as efficacy-related proxies.

Broader syntheses have reported positive associations between efficacy-related beliefs and performance, but their scope and applicability differ. Moritz et al. (2000) [7] was retained as a broad foundational synthesis, although its heterogeneous evidence base included laboratory and motor-performance tasks and was not used as direct gymnastics evidence. Lochbaum et al. (2023) [11], which restricted eligibility to pre-event self-efficacy in sport settings, is more directly relevant to the present question. Neither pooled result was applied quantitatively to artistic gymnastics, where performance is shaped by apparatus-specific demands, technical difficulty, judging procedures, maturation, training history, and prior performance. Confidence-related beliefs may affect performance through effort regulation and persistence [14,17], while challenge–threat appraisals and emotional interpretation may influence attentional control under pressure [18]. However, these mechanisms have rarely been measured directly in gymnastics-specific studies. For this reason, evidence from artistic gymnastics needs to be evaluated not only in terms of whether an association is reported but also in terms of construct specificity, sample characteristics, timing of assessment, and the type of performance outcome used.

Despite the theoretical relevance of self-efficacy for artistic gymnastics, research addressing this construct within the sport has developed in a relatively dispersed manner. Contributions have emerged from different lines of sport psychology research, often focusing on related constructs such as confidence, competitive anxiety, perceived physical ability, or performance expectations. As a result, the available evidence is heterogeneous and has not been systematically organized according to whether studies assessed direct task-specific self-efficacy, broader confidence-related constructs, or indirect perceived-ability measures. This limits the extent to which firm conclusions can be drawn about the role of self-efficacy in artistic gymnastics performance.

Therefore, the aim of this systematic review was to synthesize and critically evaluate the evidence on the relationship between self-efficacy and performance in artistic gymnastics while distinguishing direct task- or apparatus-specific efficacy measures from efficacy-related proxies based on performance predictions or adapted task-specific confidence measures. Related confidence constructs were considered only when they met the eligibility criteria or when they clarified construct boundaries. Particular attention was given to assessment timing and specificity, sample characteristics and competitive level, and the type of performance outcome.

## 2. Materials and Methods

**Figure 1:**
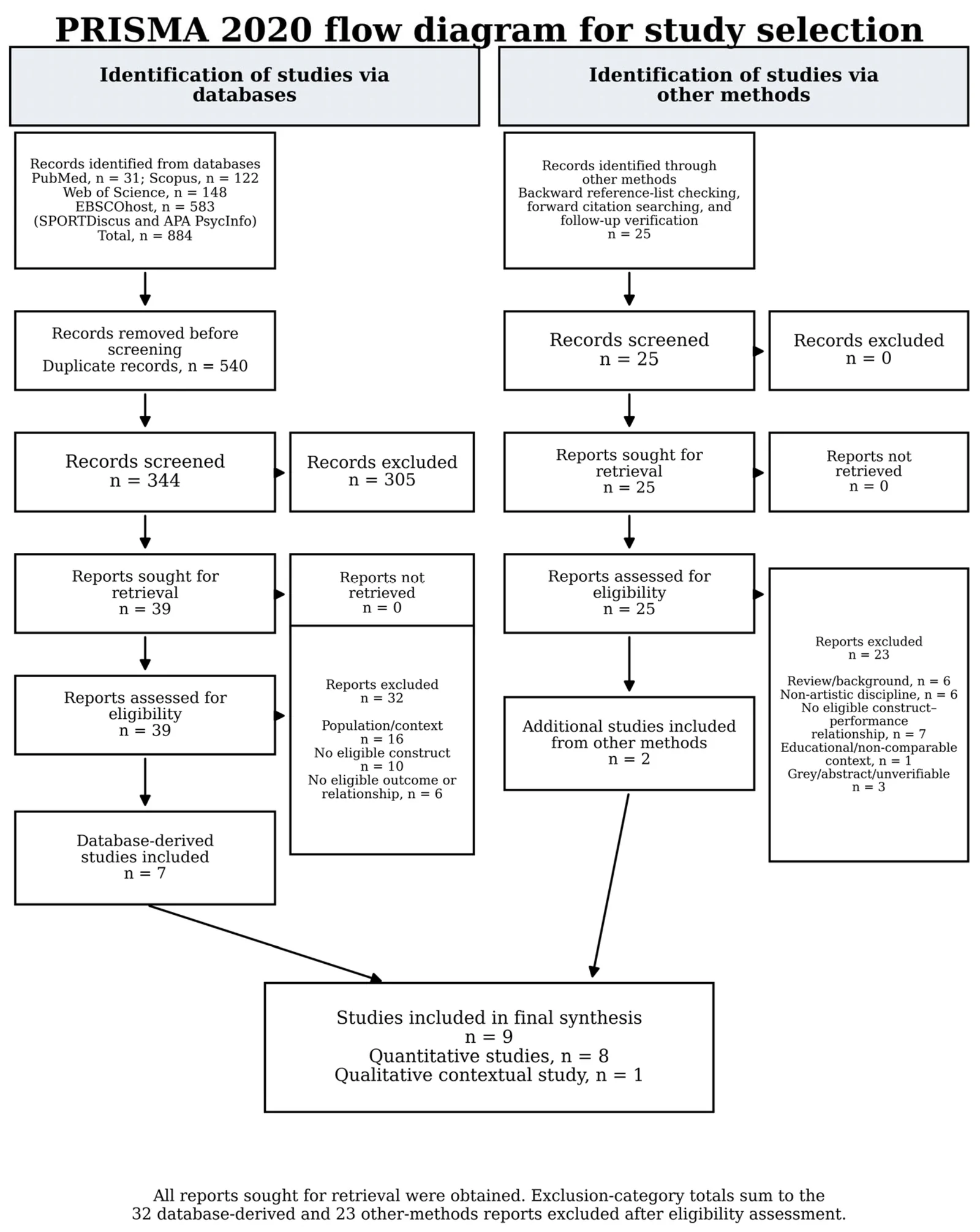
PRISMA 2020 flow diagram of study selection. All reports sought for retrieval were obtained. The final synthesis included nine studies: eight quantitative studies and one qualitative contextual study.

This systematic review was conducted and reported in accordance with the Preferred Reporting Items for Systematic Reviews (PRISMA) 2020 guidelines [19] and the PERSIST (PRISMA in Exercise, Rehabilitation, Sport Medicine and Sports Science) 2021 guidance [20]. The completed PRISMA 2020 checklist is provided as Supplementary File S1. The review protocol was prospectively registered in the PROSPERO database (registration number: CRD420251266838). The registered record also described an exploratory pilot assessment; the present report addresses only the systematic-review component. During peer review, supplementary backward reference-list checking and forward citation searching were expanded, two eligible reports were added, and competitive, educational, and qualitative evidence were separated explicitly in the synthesis. These refinements improved search completeness and interpretive transparency without changing the primary review question; they were not entered as formal amendments in PROSPERO. Full electronic search strategies for all databases are reported in Supplementary Table S1. Records from the other-methods route are documented in Supplementary Table S2 and Figure 1.

### 2.1. Eligibility Criteria

Eligibility criteria were defined according to the Population, Exposure, Comparator, Outcome, and Study Design (PECOS) framework. Two population contexts were eligible but were synthesized separately: (i) developmental or competitive artistic gymnasts engaged in structured training or competition, which constituted the primary evidence; and (ii) educational or university cohorts completing explicitly artistic gymnastics skills or routines under structured instruction with a formal performance assessment, which constituted indirect evidence. Competitive athletes were primarily classified within Tiers 2–5 according to McKay et al. [21]. Educational and university-based cohorts were not combined with competitive artistic gymnastics samples because their task demands, participant characteristics, and performance-evaluation procedures differed from formal competitive contexts.

Studies were eligible if they assessed self-efficacy in an artistic gymnastics context, task- or apparatus-specific efficacy expectations, or a prespecified efficacy-related proxy (a performance prediction or a confidence measure explicitly adapted or operationalized for a specific gymnastics task) and examined the measure in relation to a performance context or outcome. Measures were classified as direct self-efficacy, task- or apparatus-specific efficacy expectations, performance-prediction proxies, adapted task-specific confidence proxies, broader confidence or perceived-ability measures, or qualitative contextual evidence. Broader self-confidence, anxiety-subscale, or perceived-ability records without an eligible task-specific construct–performance relationship were excluded from the main corpus and documented in Supplementary Table S2. One qualitative study that explicitly explored self-efficacy in gymnastics was retained solely for contextual interpretation.

Where available, comparator conditions included contrasts between athletes with higher versus lower levels of self-efficacy, differences across competitive level or sample type, as well as comparisons involving concurrent psychological states such as anxiety or fatigue.

Eligible outcomes focused on performance-related measures and were operationalized in terms of performance efficacy and performance efficiency. Performance efficacy included judged competition scores, apparatus-specific scores, execution scores, grades, error-related indicators, and discrepancies between predicted and actual performance (without treating predicted scores as direct self-efficacy measures), whereas performance efficiency encompassed indicators of movement quality within routines or biomechanical markers such as kinematic stability or center-of-mass displacement, when available. Studies that assessed psychological constructs without reporting a performance-related outcome were not included in the main synthesis.

Observational study designs (cross-sectional and longitudinal) were included, as were acute assessments in training or competition. Experimental or quasi-experimental studies were eligible when they examined changes in self-efficacy or a prespecified task-specific efficacy-related proxy together with performance-related outcomes in artistic gymnastics tasks. Within PECOS, “Exposure” denoted the assessment or manipulation of these constructs. Studies had to be full-text, peer-reviewed articles in English; no age or gender restriction was applied. Grey literature, including theses and conference abstracts, was excluded because it did not meet the prespecified publication criterion.

### 2.2. Exclusion Criteria

Studies were excluded if they did not involve an artistic gymnastics task, skill, or routine that met the population and performance criteria above. Non-competitive status alone was not an exclusion criterion; educational or recreational records were excluded when the task or outcome was insufficiently gymnastics-specific, when no eligible construct–performance relationship was reported, or when the context could not support even indirect interpretation. Studies involving rhythmic gymnastics, TeamGym, acrobatic gymnastics, trampoline, general physical education, or other non-artistic gymnastics activities were excluded unless artistic gymnastics data were clearly separable and all other criteria were met. Studies were also excluded if they did not assess self-efficacy or an eligible task-/performance-specific construct, did not report a performance-related outcome or direct construct–performance analysis, or focused exclusively on injury prevention, rehabilitation, or clinical populations. Finally, articles were excluded if they were not full-text, peer-reviewed papers in English. Reviews and meta-analyses were not primary studies but were used for contextualization and backward reference-list checking.

### 2.3. Outcome Operationalization and Synthesis Framework

To support a coherent interpretation of performance outcomes, the Multi-Action Plan (MAP) model proposed by Bertollo et al. [22] was adopted as an analytical framework. Within this framework, performance outcomes were classified a priori into efficacy- and efficiency-related dimensions. Performance efficacy outcomes reflected the accuracy or success of task execution (e.g., judged execution or all-around scores, apparatus-specific error rates, discrepancies between predicted and actual performance), whereas performance efficiency outcomes reflected qualitative or biomechanical aspects of execution (e.g., expert-rated movement quality or kinematic indicators of movement control), when available.

The MAP framework was used as a heuristic tool to organize performance-related outcomes rather than as a model directly tested by the included studies. The efficacy–efficiency distinction was retained as a conceptual framework to guide the interpretation of performance outcomes and to identify gaps in the available literature. However, because most included studies reported efficacy-related outcomes, such as judged scores, execution scores, or performance results, efficiency-related indicators were considered only when explicitly available. When a performance score could reflect both successful task completion and technical quality, the outcome was classified according to the primary way it was operationalized in the original study.

Given the substantial methodological heterogeneity across studies in terms of self-efficacy assessment, performance outcomes, and study design, findings were synthesized narratively rather than by meta-analysis. The narrative synthesis was informed by Synthesis Without Meta-analysis (SWiM) reporting principles [23], with studies grouped by construct specificity, sample context, study design, and performance outcome. When efficiency-related indicators were absent, studies were interpreted primarily within the efficacy dimension. Two structured narrative sensitivity checks were conducted: the first considered studies explicitly measuring direct self-efficacy measures, gymnastics task-/apparatus-specific efficacy expectations, or prespecified efficacy-related performance-prediction proxies; the second separated competitive artistic gymnastics samples from educational or non-competitive samples.

### 2.4. Sources and Search Strategy

An electronic literature search was conducted across PubMed (National Library of Medicine interface), Scopus (Elsevier), Web of Science Core Collection (Clarivate), and EBSCOhost (SPORTDiscus and APA PsycInfo). APA PsycInfo is reported using its current database name; the registered protocol and retained search documentation used the historical label ‘PsycLIT’. The database searches covered records from inception to 18 December 2025 and were supplemented by backward reference-list checking of relevant reviews and meta-analyses and forward citation searching from eligible or candidate records. The designated review sources and candidate records in the other-methods route were last checked on 26 July 2026. Bandura’s 1977 publication [13] was used solely as the conceptual reference for defining self-efficacy and was not treated as an included study.

The search strategy was developed according to the PECOS framework. Population-related terms included “artistic gymnastics”, “competitive gymnastics”, and “gymnastics”, as well as the truncated term “gymnast*” to capture possible variations in terminology across studies. Terms related to the psychological construct included both “self-efficacy” and “self efficacy”, as well as related confidence constructs. Outcome-related terms were selected to capture performance-related indicators consistent with the conceptual distinction between efficacy and efficiency and included “performance”, “error rates”, “execution scores”, “event score”, “execution errors”, and “kinematic stability”. The complete search strings used for each database are reported in Supplementary Table S1.

Comparator elements and study design criteria were not incorporated into the electronic search strings because their inclusion would have substantially reduced search sensitivity. Instead, these criteria were applied during the screening and eligibility phases in accordance with PRISMA 2020 recommendations [19]. Boolean operators were used to combine search terms, and searches were limited to title, abstract, and keyword fields when available to improve specificity.

Database-specific syntax was adapted as required. The search strategies retained in the review files are reported in Supplementary Table S1. The EBSCOhost export preserved only the combined SPORTDiscus/APA PsycInfo yield; separate database yields and any unavailable platform-specific line histories were not reconstructed post hoc. Following database screening, backward checking was conducted on the reference lists and included-study tables of five prior reviews and meta-analyses: Moritz et al. (2000) [7], Craft et al. (2003) [9], Lochbaum et al. (2022) [10], Lochbaum et al. (2023) [11], and Jekauc et al. (2025; first published online in 2023) [12]. These sources were used for checking and contextualization only. The complete other-methods route—backward reference-list checking of these five reviews, forward citation searching, and follow-up verification of candidate records—yielded 25 records. Each record, its identification note, and its eligibility decision are reported in Supplementary Table S2; aggregate counts are shown in Figure 1. No meta-analysis was conducted in the present review.

No formal assessment of reporting bias (e.g., publication bias or small-study effects) was conducted, as the limited number of included studies and the absence of quantitative synthesis precluded meaningful evaluation. The feasibility of meta-analysis was assessed after extraction of available effect estimates. A quantitative synthesis was not conducted because too few studies reported statistically comparable effects for the same construct–outcome relationship, and several studies reported multiple non-independent apparatus-specific estimates. Reference management and bibliography organization were performed using Zotero (version 7.0.22), while title/abstract and full-text screening were conducted using Rayyan [24], as illustrated in Figure 1.

### 2.5. Data Collection Process

All records retrieved from the electronic searches were imported into the Rayyan web-based systematic review software [24]. Duplicate records identified across databases were automatically detected and removed within the platform prior to the screening process. Records identified through backward reference-list checking, forward citation searching, and follow-up verification were also screened against the same eligibility criteria and were documented separately in Supplementary Table S2. Rayyan was subsequently used to support title and abstract screening and to manage the study selection workflow.

Two independent reviewers (F.M. and G.D.P.) screened all retrieved records in a two-stage selection process. In the first stage, titles, abstracts, and keywords were independently examined according to the predefined inclusion and exclusion criteria. Articles deemed potentially eligible by at least one reviewer were advanced to the second stage. In the second stage, full-text articles were independently assessed for eligibility by the same two reviewers. Any disagreements or uncertainties arising at either screening stage were resolved through discussion; when consensus could not be reached, a third reviewer (D.C.) was consulted to adjudicate and reach a final decision. The entire screening and selection process including the other-methods checking was conducted in accordance with PRISMA 2020 recommendations [19].

### 2.6. Data Items

For each included study, data were extracted regarding geographical location, study design, sample characteristics (including sample size, age, and gender), sample context and competitive level, self-efficacy assessment methods, construct category, instrument status, and performance assessment approaches, including efficacy- and/or efficiency-related indicators. Constructs were categorized as direct self-efficacy, task- or apparatus-specific efficacy expectations, performance-prediction proxies, adapted task-specific confidence proxies, broader confidence/perceived-ability measures, or qualitative contextual evidence. Instrument status was coded as validated, adapted, researcher-developed, non-standardized, or qualitative interview-based, according to the information reported in each study. F.M. and G.D.P. extracted and organized the data in structured tables; entries and reported statistics were then checked against the source reports during preparation of Table 1 and Table S3, with discrepancies resolved by discussion. Data extraction was collaborative and was not performed independently in duplicate. The authors did not contact study investigators and did not use automation tools for data extraction.

When explicitly reported, information regarding the specific apparatus was also extracted. In addition, the main findings relevant to the association between self-efficacy or efficacy-related beliefs and performance were recorded. Where available, effect estimates and statistical information were extracted, including correlations, beta coefficients, explained variance, *p*-values, group comparisons, and confidence intervals. When effect estimates were not reported or could not be derived from the available information, this was coded as not reported rather than inferred. When sample size information was not explicitly reported in the original studies, this was noted and considered within the risk of bias assessment rather than inferred. Each study was also assigned to a synthesis category: competitive artistic gymnastics evidence, experimental evidence, educational/non-competitive evidence, qualitative contextual evidence, or excluded/background evidence when identified through supplementary backward reference-list checking or forward citation searching.

### 2.7. Risk of Bias Assessment

Risk of bias was assessed independently by two reviewers (F.M. and G.D.P.). Randomized controlled trials were evaluated using the Cochrane Risk of Bias 2 (RoB 2) tool [31], while observational and cross-sectional studies were assessed using the National Institutes of Health (NIH) Quality Assessment Tool for Observational Cohort and Cross-Sectional Studies [32]. The NIH tool was selected because most non-randomized quantitative studies were observational, cross-sectional, or cohort-like designs rather than non-randomized intervention studies estimating causal effects. Therefore, ROBINS-I was not considered the most appropriate tool for the majority of included quantitative studies.

The majority of included studies were quantitative. One qualitative study explored self-efficacy beliefs in gymnastics contexts and was retained because it provided contextual information on gymnastics-specific sources of self-efficacy and performance-related behaviors [27]. However, because it did not provide quantitative associations between self-efficacy and performance outcomes, it was used only for descriptive and contextual interpretation and was not included in the synthesis of quantitative findings. Accordingly, this qualitative study was formally appraised using the Critical Appraisal Skills Programme (CASP) Qualitative Studies Checklist [33]. The appraisal focused on the clarity of the research aims, appropriateness of the qualitative methodology, recruitment strategy, data collection, reflexivity, ethical considerations, rigor of data analysis, clarity of findings, and relevance to the review question. CASP results are reported in the Supplementary Materials.

For randomized trials, bias was evaluated across the five standard RoB 2 domains (randomization process, deviations from intended interventions, missing outcome data, measurement of the outcome, and selection of the reported result), and an overall judgment of “low risk,” “some concerns,” or “high risk” was assigned according to the tool guidance [31]. For observational studies, methodological quality was rated as “good,” “fair,” or “poor” according to the NIH assessment criteria [32]. For the qualitative study, CASP items were summarized narratively rather than converted into a numerical score, in line with the purpose of the tool. Any disagreements between reviewers were resolved through discussion until consensus was reached.

In addition to risk of bias, the certainty of the overall body of evidence was summarized narratively by considering study design, risk of bias, inconsistency, indirectness, imprecision, and reporting limitations. Because no meta-analysis was conducted and the evidence base was small and heterogeneous, certainty descriptors were used to support cautious interpretation rather than pooled quantitative conclusions. The labels “low” and “very low” were descriptive narrative judgments and should not be interpreted as formal GRADE ratings.

## 3. Results

### 3.1. Study Selection

Seven studies were retained through the database-search route and two through other methods, yielding nine studies in the final synthesis: eight quantitative studies and one qualitative contextual study. Figure 1 reports the full identification, screening, and exclusion counts; record-level decisions for the other-methods route are provided in Supplementary Table S2.

### 3.2. Characteristics of Included Studies

The nine studies included in the systematic review were conducted across diverse geographical contexts, including North America, Europe, Asia, and Oceania. The evidence base included studies conducted in competitive artistic gymnastics, experimental gymnastics-specific settings, educational or university-based gymnastics contexts, and one qualitative contextual investigation. A summary of the main characteristics of the included studies, including sample information, instrument type, assessment timing, performance outcome, and available statistics, is provided in Table 1.

Sample sizes ranged from 10 to 370 participants and included gymnasts or gymnastics participants spanning developmental, club-level, collegiate, youth competitive, and educational or university-based cohorts. Competitive samples were distinguished from educational or non-competitive samples throughout the synthesis, because the latter could not be assumed to represent the same competitive demands, performance constraints, or athlete-caliber characteristics as artistic gymnasts classified within McKay et al.’s Tiers 2–5. One study employed a randomized controlled design examining the effects of self-modeling versus a control condition on balance beam performance [4], whereas the remaining quantitative studies adopted observational, correlational, longitudinal, or educational designs. One qualitative study was retained only for contextual interpretation of gymnastics-specific sources of self-efficacy and performance-related behaviors.

With respect to apparatus specificity, two studies focused on single-apparatus performance, targeting the balance beam [4] and the uneven bars [5], respectively. Several studies reported apparatus- or event-specific findings across multiple artistic gymnastics events, including vault, bars, beam, floor, or all-around performance, although the number and type of apparatus differed across studies. One qualitative investigation examined self-efficacy-related experiences in relation to gymnastics skills, fear of injury, and performance-related behaviors [27]. No study provided sufficient evidence to establish a stable apparatus-specific hierarchy of self-efficacy effects across the sport.

Self-efficacy and related constructs were assessed using heterogeneous approaches. Measures were classified as direct task- or apparatus-specific self-efficacy; efficacy-related proxies based on performance expectations or predicted scores; adapted task-specific confidence proxies; broader sport confidence, self-confidence, or perceived-ability measures; or qualitative contextual evidence. Earlier investigations primarily relied on task- or apparatus-specific performance predictions elicited before competition and treated here as efficacy-related proxies, without the use of standardized instruments [5,16,25,26]. Other studies used researcher-developed task-specific self-efficacy instruments, validated general physical self-efficacy measures, or an adapted task-specific confidence measure. Because these instruments differed substantially in construct specificity and validation status, neither the performance-prediction nor adapted-confidence proxies were treated as equivalent to direct self-efficacy measures.

Across studies, performance outcomes were predominantly operationalized using official judged scores, competition results, event-specific scores, all-around scores, or structured expert evaluations. Educational studies used practical examination grades or expert-rated routine segments rather than official competitive scores and were therefore synthesized separately from competitive artistic gymnastics studies. Variation in assessment timing, samples, constructs, and outcomes supported narrative rather than quantitative synthesis.

### 3.3. Risk of Bias

**Figure 2:**
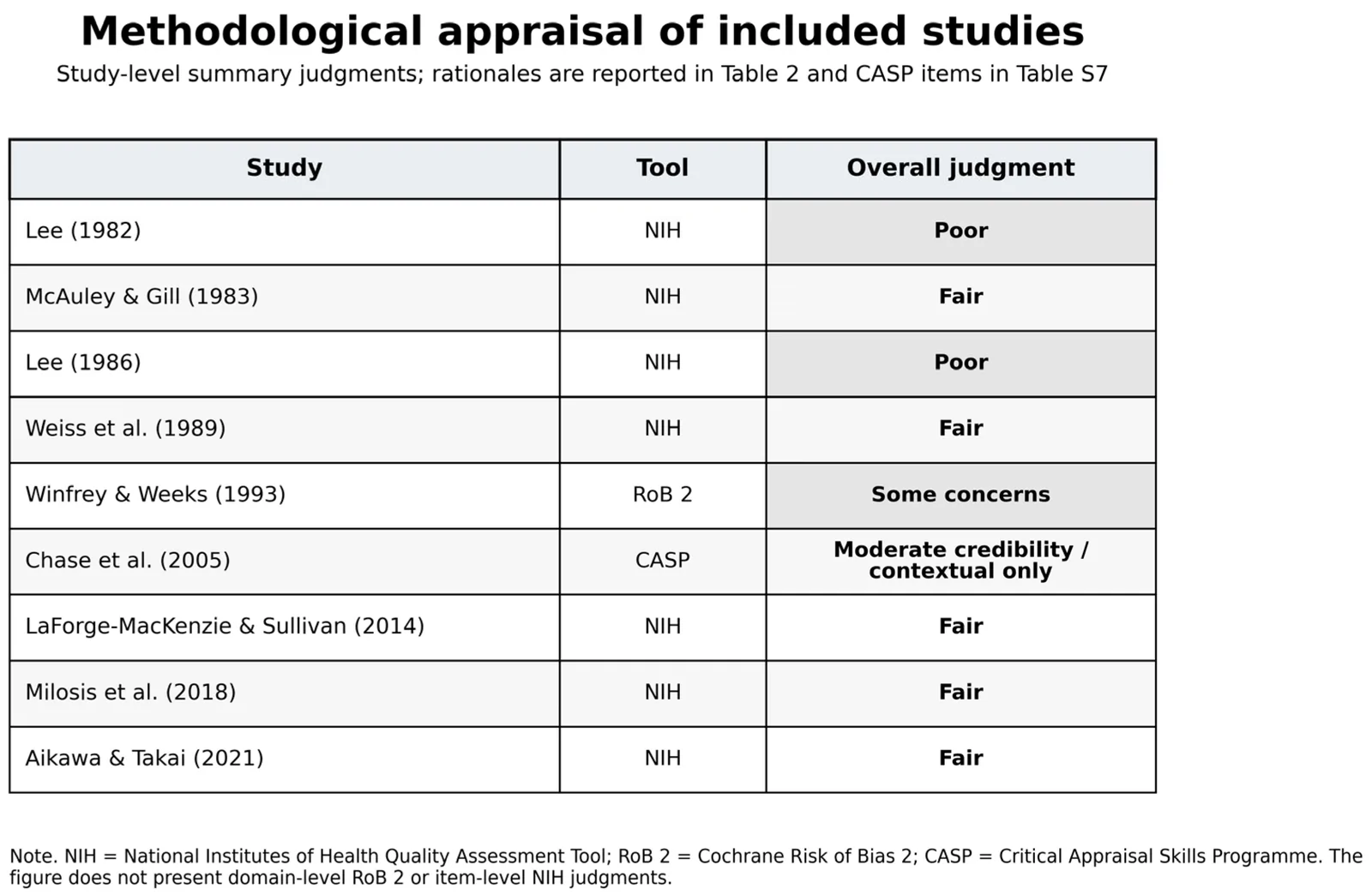
Methodological appraisal of included studies. Summary judgments are shown by study. Study-level rationales are reported in Table 2, and the item-level CASP appraisal of the qualitative study is reported in Supplementary Table S7. The figure does not present domain-level RoB 2 or item-level NIH judgments. NIH = National Institutes of Health Quality Assessment Tool; RoB 2 = Cochrane Risk of Bias 2; CASP = Critical Appraisal Skills Programme [4,5,16,25,26,27,28,29,30].

Risk of bias was assessed using the Cochrane Risk of Bias 2 (RoB 2) tool for randomized controlled trials and the National Institutes of Health (NIH) Quality Assessment Tool for Observational Cohort and Cross-Sectional Studies for observational designs. The qualitative study was formally appraised using the Critical Appraisal Skills Programme (CASP) Qualitative Studies Checklist [33] and was retained for contextual interpretation only. The overall results of the methodological appraisal are summarized in Figure 2.

The methodological quality of the included studies was primarily limited by selection- and confounding-related issues. The single randomized controlled trial [4] presented some risk-of-bias concerns, mainly due to incomplete reporting of the randomization process and small group sizes. In contrast, deviations from intended interventions, outcome measurement, and outcome completeness were judged to be of lower concern, and selective outcome reporting was considered unlikely.

Among the non-randomized studies assessed using the NIH Quality Assessment Tool, study-level judgments ranged from fair to poor methodological quality. Common limitations included the use of convenience samples, single-club or single-course recruitment, educational or university-based cohorts, limited representativeness, and modest sample sizes. Additionally, key confounding factors—such as prior ability, previous performance, age, maturation, competitive level, training load, fatigue, injury status, and coaching influences—were rarely measured or statistically controlled.

Additional methodological limitations concerned the assessment of self-efficacy and related confidence constructs across studies. Earlier investigations relied on context-specific and non-standardized indicators, including apparatus-specific predicted scores [5,16,25,26]. Although conceptually aligned with self-efficacy theory when tied to specific gymnastics tasks, predicted scores were treated as efficacy-related proxies rather than as direct measures of self-efficacy; they also lacked formal standardization and psychometric validation. The confidence measure adapted for the balance beam [4] was classified in the same way. General physical self-efficacy scales, sport confidence inventories, self-confidence subscales, and perceived physical ability measures were treated as related but non-equivalent constructs. Only task- or apparatus-specific self-efficacy instruments and ratings were interpreted as direct evidence on self-efficacy.

Outcome measurement was generally stronger when studies relied on official judged event scores, all-around scores, or competition results. However, performance outcomes were not homogeneous across studies: some reflected official competitive performance, others reflected structured expert grading in educational contexts, and one qualitative study did not provide quantitative performance outcomes. Sample size justification was rarely reported, while missing data and attrition were minimal and transparently documented when present. A detailed study-level methodological appraisal is reported in Table 2.

### 3.4. Narrative Synthesis

The included studies demonstrated substantial heterogeneity in construct operationalization, performance outcomes, timing of assessment, participant characteristics, and study design.

Consequently, a meta-analysis was not conducted. Instead, available study-level statistics are reported in Table 1 and Supplementary Table S3, and findings were synthesized narratively according to construct specificity, sample context, study design, and performance outcome. This approach was used because the available studies differed in construct operationalization, apparatus, timing, performance outcome, and statistical reporting, and because several apparatus-specific estimates were non-independent within studies.

#### 3.4.1. Self-Efficacy Measurement Approaches

Across the included studies, direct self-efficacy measures and efficacy-related proxies were assessed through diverse approaches, including task- and apparatus-specific self-efficacy ratings or inventories, performance predictions, an adapted task-specific confidence measure, skill-specific self-efficacy questionnaires, and qualitative interviews. Performance predictions and the adapted confidence measure were treated as proxies rather than as measures equivalent to direct self-efficacy.

Three early competitive studies used performance expectations or predicted scores as task- or apparatus-specific efficacy-related proxies. In these studies, gymnasts estimated expected performance at varying temporal distances from competition. Novice female gymnasts provided predictions before competition [25], club-level athletes reported expected uneven bars scores [5], and youth male gymnasts expressed event-by-event expectations shortly before competition [26]. These proxies were closely tied to upcoming performance tasks and therefore conceptually aligned with self-efficacy theory, but they were not treated as equivalent to standardized or validated direct self-efficacy instruments.

McAuley and Gill [16] assessed general physical self-efficacy and task-specific efficacy inventories in collegiate female gymnasts, while predicted event scores were treated separately as efficacy-related proxies. This design allowed comparison among broader perceived physical capability, direct event-specific efficacy beliefs, and predicted-score proxies.

In educational contexts, two studies relied on task-specific self-efficacy ratings rather than standard competitive inventories. Milosis et al. [29] assessed apparatus-specific self-efficacy longitudinally across an academic year and related these ratings to practical examination grades. LaForge-MacKenzie and Sullivan [28] employed moment-to-moment verbal self-efficacy ratings collected immediately before each mandatory skill within a single continuous routine, pairing these ratings with expert evaluations of body control. Because these studies involved educational or university-based learning contexts rather than competitive artistic gymnastics, their findings were interpreted as indirect evidence.

Winfrey and Weeks [4] used a balance beam confidence inventory adapted from the State Sport Confidence Inventory and operationalized for the gymnastics task. Because it was adapted rather than independently validated as a gymnastics-specific self-efficacy scale, it was classified as an efficacy-related proxy, not as a direct measure equivalent to self-efficacy. Other sport confidence, CSAI-based self-confidence, and perceived-ability records were likewise not treated as task-specific self-efficacy.

#### 3.4.2. Competitive Artistic Gymnastics Evidence

Across competitive artistic gymnastics studies using direct task-specific efficacy measures or prespecified task-/event-specific efficacy-related proxies, the evidence generally suggested positive associations between specific efficacy-related beliefs and performance. However, the strength and consistency of these associations varied across studies, apparatus, and measurement approaches.

In novice female gymnasts, athletes’ predicted apparatus scores, treated as efficacy-related proxies, were associated with actual all-around performance and appeared to provide information beyond prior competition results, although coach expectancy emerged as the strongest predictor [25]. This suggests that athletes’ task-specific predictions may be relevant to performance prediction, but also that external behavioral or observational indicators may offer stronger predictive information in some contexts.

In club-level athletes performing on uneven bars, an apparatus-specific expected-score proxy was associated with competition scores, but contemporaneous training execution emerged as the most accurate predictor of competitive performance [5]. This efficacy-related proxy may provide complementary information about readiness, but should not be interpreted as a substitute for behavioral indicators of preparation or recent training quality.

In youth male gymnasts, event-specific expected-score proxies were positively associated with event and all-around performance, with associations varying by apparatus [26]. However, because apparatus-specific estimates within the same study are not statistically independent, these findings were interpreted as evidence of task specificity rather than as proof that self-efficacy is consistently stronger for one apparatus than another.

The study by McAuley and Gill [16] further supported the importance of construct specificity. Task-specific efficacy was associated with official event scores for vault (r = 0.284), beam (r = 0.582), bars (r = 0.717), and floor (r = 0.426), whereas general physical self-efficacy was weaker and less consistent. This pattern supports Bandura’s assumption that efficacy beliefs are most informative when measured at a level of specificity closely matched to the task and outcome being predicted.

Aikawa and Takai [30] provided more cautious evidence. In a first-division university gymnastics club, self-efficacy was assessed 5 days before an internal selection match and was only weakly related to performance (r = 0.22), whereas confidence (r = 0.32) and goal imagery (r = 0.43) showed clearer associations with performance. This study therefore tempers the conclusion that self-efficacy is uniformly or strongly associated with gymnastics performance across all competitive contexts.

#### 3.4.3. Experimental Evidence

The only randomized controlled trial examined the effects of self-modeling on balance beam performance and an adapted balance beam confidence measure treated as an efficacy-related proxy [4]. No significant between-group differences in the proxy measure or performance were reported. The main positive finding was stronger calibration between self-rated and actual post-test performance in the self-modeling group (r = 0.92) than in the control group (r = 0.02). Given the sample size (n = 11) and incomplete reporting of the randomization process, this study does not provide strong causal evidence that self-modeling improves efficacy-related beliefs or performance.

#### 3.4.4. Educational and Non-Competitive Evidence

In educational and university-based settings, self-efficacy was examined in relation to gymnastics learning, routine execution, or practical examination performance rather than official competitive outcomes. Milosis et al. [29] reported positive associations between skill- or apparatus-specific self-efficacy and practical gymnastics grades across an academic year (males r = 0.46–0.65; females r = 0.54–0.63). These findings suggest that self-efficacy may be relevant to gymnastics skill learning and assessment in structured educational contexts, but they cannot be directly generalized to competitive artistic gymnastics.

LaForge-MacKenzie and Sullivan [28] examined moment-to-moment self-efficacy during a continuous educational gymnastics routine. Immediate self-efficacy ratings did not significantly predict the immediately subsequent performance segment, and performance did not consistently predict subsequent self-efficacy at the same short time scale. This finding suggests that very proximal, momentary efficacy ratings may not translate directly into immediate performance changes within uninterrupted novice routines.

Taken together, educational studies provide useful indirect information on the relationship between perceived capability and gymnastics task execution. However, because participants were not competitive artistic gymnasts and performance outcomes were educational grades or expert-rated routine segments, these studies were not used as primary evidence for conclusions about competitive artistic gymnastics performance.

#### 3.4.5. Qualitative Contextual Evidence

One qualitative study explored self-efficacy beliefs in gymnastics contexts and was retained as contextual evidence [27]. This study highlighted gymnastics-specific sources of efficacy beliefs, including mastery experiences, fear of injury, perceived readiness, and the role of psychological strategies in managing demanding skills. Because it did not provide quantitative associations between self-efficacy and performance outcomes, it was not included in the quantitative synthesis and did not contribute effect estimates.

The qualitative findings were used to contextualize how self-efficacy may operate in artistic gymnastics, particularly in relation to fear, injury-related concerns, and confidence in performing complex skills. However, they do not establish the magnitude or direction of the relationship between self-efficacy and judged performance.

#### 3.4.6. Context, Apparatus, and Performance Outcomes

Task demands and competitive context appeared to influence the relationship between efficacy-related beliefs and performance. Studies using apparatus- or event-specific direct measures or prespecified efficacy-related proxies generally provided more directly interpretable findings than studies using broad physical self-efficacy, general sport confidence, or educational perceived-ability measures. Apparatus-specific findings suggested that efficacy-related beliefs may vary across events, but the available evidence is insufficient to establish robust apparatus-specific conclusions or rankings.

Most included studies reported efficacy-related performance outcomes, such as judged scores, competition results, grades, or discrepancies between predicted and actual performance. Efficiency-related indicators, such as biomechanical measures or kinematic stability, were rarely reported. Therefore, the MAP efficacy–efficiency distinction was useful for organizing outcomes and identifying gaps, but the available literature mainly addressed performance efficacy rather than performance efficiency.

Overall, the findings suggest that task-specific efficacy-related beliefs may be positively associated with artistic gymnastics performance, particularly when either direct measures or prespecified proxies are closely matched to the apparatus, skill, or performance outcome. However, this association is limited by small samples, heterogeneous construct definitions, variable measurement timing, limited control of confounding, and the inclusion of indirect educational or confidence-related evidence. The results should therefore be interpreted as low-certainty evidence of a positive but context-dependent association rather than as evidence of a consistent or causal effect.

## 4. Discussion

The present systematic review examined the association between self-efficacy, prespecified efficacy-related proxies, and performance in artistic gymnastics, and evaluated how direct and proxy measures have been used across competitive and non-competitive gymnastics contexts. Overall, the findings suggest a positive but heterogeneous and low-certainty association between task- or apparatus-specific efficacy-related beliefs and performance in artistic gymnastics. The most directly applicable evidence came from competitive studies using direct task-specific efficacy measures or task-/event-specific performance-prediction proxies together with judged performance outcomes [5,16,25,26,30]. However, the strength and consistency of this association varied across samples, apparatus, timing of assessment, and measurement approaches.

In several studies, task- or event-specific performance-prediction proxies demonstrated greater predictive value than historical performance indicators [25,26]; however, this pattern was not uniform across the evidence base. In one study, contemporaneous training execution emerged as the strongest predictor of competitive outcomes, exceeding the predictive contribution of an expected-score proxy [5]. Taken together, these findings suggest that efficacy-related information may complement, rather than replace, behavioral indicators derived from training execution.

From a psychological perspective, self-efficacy can be conceptualized as a proximal belief construct that shapes how gymnasts approach performance demands, particularly under evaluative pressure. Athletes with higher task-specific self-efficacy may be more likely to interpret competitive situations as manageable challenges rather than threats, thereby supporting attentional control, adaptive effort regulation, and emotional stability [14,34]. Conversely, low self-efficacy may be associated with cognitive anxiety, disrupted motor planning, or increased performance variability, especially in sports such as artistic gymnastics that are characterized by high precision requirements and limited tolerance for error [9]. Nevertheless, the included studies rarely measured these mechanisms directly; therefore, they should be interpreted as theoretically plausible explanations rather than established causal pathways in artistic gymnastics.

These dynamics align with broader psychobiosocial models of performance, which conceptualize athletic outcomes as emerging from continuous interactions between psychological states, biological readiness, and contextual demands [35]. Within this framework, self-efficacy may operate as a dynamic construct that both influences and is shaped by performance-related experiences. Successful execution and positive feedback may strengthen efficacy beliefs through mastery experiences, whereas repeated errors, injuries, or negative evaluations may undermine confidence and affect subsequent performance attempts. However, most included studies were cross-sectional, observational, or based on short-term assessments; consequently, reciprocal or longitudinal relationships between self-efficacy and performance remain insufficiently tested in artistic gymnastics.

A major issue identified by this review concerns the heterogeneity of self-efficacy measurement approaches. Earlier studies predominantly relied on unstandardized, apparatus- or event-specific performance predictions assessed proximal to competition [5,16,25,26]. These predictions were conceptually aligned with Bandura’s task-specific formulation when closely tied to an upcoming gymnastics task, but they were treated as efficacy-related proxies rather than direct self-efficacy measures because they generally lacked formal standardization and psychometric validation. McAuley and Gill [16] provided particularly useful evidence by distinguishing broader physical self-efficacy, task-specific efficacy inventories, and predicted event scores.

Other investigations used an adapted task-specific confidence measure as an efficacy-related proxy [4], researcher-developed task-specific self-efficacy instruments [29,30], and a validated general physical self-efficacy measure [16]. These approaches varied in task specificity and validation status and were not treated as interchangeable. Direct task- or apparatus-specific self-efficacy measures provided the closest test of self-efficacy theory; performance-prediction and adapted-confidence measures provided proxy evidence, while broader confidence or perceived-ability measures provided indirect evidence.

The synthesis also distinguished competitive artistic gymnastics evidence from educational or non-competitive gymnastics contexts. Educational studies provided useful information about perceived capability and gymnastics skill learning, particularly when skill-specific self-efficacy was related to practical examination grades or routine-segment performance [28,29]. However, these findings cannot be directly generalized to competitive artistic gymnastics, because participants, performance demands, scoring procedures, and athlete-caliber characteristics differed from formal competitive settings. Accordingly, educational and non-competitive studies were interpreted as sensitivity or indirect evidence rather than as primary evidence for competitive artistic gymnastics performance.

Interpretation of the findings should also consider methodological limitations of the available evidence. Across studies, designs varied considerably in terms of sampling strategies, sample size, competitive level, and the extent to which contextual and athlete-related factors—such as prior ability, previous performance, age, maturation, training exposure, injury status, fatigue, training load, and coaching environment—were measured or controlled. These limitations reduce the certainty of the evidence and prevent strong conclusions about causality, directionality, or generalizability across competitive levels. Despite these limitations, performance outcomes were often assessed using official judged scores, competition results, or structured expert ratings, which strengthens confidence in the relevance of the performance indicators used. However, the heterogeneity of these outcomes means that they should not be treated as interchangeable.

The application of the efficacy–efficiency distinction highlighted an important gap in the literature. Although most included studies assessed performance through efficacy-related indicators, such as judged scores, competition results, expert ratings, or discrepancies between predicted and actual performance, few studies provided direct information on efficiency-related outcomes, including movement quality, kinematic stability, or biomechanical control. Therefore, the efficacy–efficiency framework should be interpreted primarily as a conceptual structure for organizing the evidence and identifying future research directions rather than as evidence that both dimensions have been equally investigated. Future work integrating psychological assessment with objective movement-quality indicators, such as kinematic, biomechanical, or workload-related measures, may help clarify whether efficacy beliefs are associated only with judged outcomes or also with the quality and stability of movement execution.

### 4.1. Certainty and Interpretation of the Evidence

The certainty of the available evidence should be considered low to very low. These are narrative descriptors rather than formal GRADE ratings. The judgment reflects the small number of included studies, the predominance of observational and exploratory designs, modest sample sizes, limited control of confounding variables, and substantial heterogeneity in construct operationalization, performance outcomes, and assessment timing. In particular, the available studies differed in whether they assessed direct task- or apparatus-specific self-efficacy, efficacy-related performance-prediction or adapted-confidence proxies, general physical self-efficacy, sport confidence, self-confidence, or perceived physical ability. These constructs are theoretically related but not interchangeable.

The narrative sensitivity checks supported this cautious interpretation. When attention was restricted to competitive artistic gymnastics studies using direct task-specific efficacy measures or prespecified task-/event-specific efficacy-related proxies, the evidence generally suggested a positive association with judged performance outcomes. However, when broader confidence-related constructs, educational/non-competitive samples, or general perceived-ability measures were considered, the evidence became less direct and more heterogeneous. Therefore, the conclusions of this review should be interpreted as indicating a limited and context-dependent association between task-specific efficacy-related beliefs and performance, rather than a robust, generalizable, or causal effect.

Because the reported estimates did not represent a common construct–outcome relationship and several apparatus-specific effects were non-independent, neither a pooled estimate nor a prediction interval was calculated.

### 4.2. Practical Implications

Task- or apparatus-specific self-efficacy ratings may complement readiness assessment, but they should be interpreted alongside recent training execution, technical consistency, injury and fatigue status, and coach observations. Low ratings may identify skills or apparatus on which an athlete feels underprepared; high ratings require calibration against observable performance. Given the low certainty of the evidence, these uses remain provisional.

## 5. Limitations

Conceptual heterogeneity is a central limitation of this review. Some studies used performance-prediction or adapted-confidence proxies, while others assessed general physical self-efficacy, sport confidence, self-confidence, or perceived physical ability rather than direct task-specific self-efficacy. Educational and university-based samples also differed from competitive artistic gymnastics in participant characteristics, task demands, and performance evaluation. Accordingly, conclusions about competitive gymnastics were based primarily on competitive or gymnastics-specific samples, while educational findings were retained only as indirect or sensitivity evidence.

The evidence base was small and predominantly observational, with modest samples and limited control of prior ability, previous performance, age, maturation, training load, fatigue, injury status, and coaching environment. Notably, five of the nine included studies were published between 1982 and 1993. Thus, despite the search being current to 18 December 2025, the gymnastics-specific evidence base remains sparse and relies substantially on older studies, underscoring the need for research using contemporary and validated measurement approaches. These limitations prevent firm causal or directional conclusions, and the scarcity of biomechanical or other efficiency-related outcomes restricted interpretation beyond judged performance. Restriction to peer-reviewed English-language articles and exclusion of grey literature may have introduced publication or language bias; the small, heterogeneous corpus also precluded formal assessment of publication bias or small-study effects. Data extraction was collaborative rather than independently duplicated. The EBSCOhost yield was retained only as a combined SPORTDiscus/APA PsycInfo total, and exact platform-specific line histories were not available for every source; missing search details were not reconstructed post hoc. Although supplementary backward reference-list checking and forward citation searching were undertaken, additional eligible studies may have been missed.

## 6. Future Directions

Future research should use adequately powered longitudinal designs to examine reciprocal relationships between task- or apparatus-specific self-efficacy and performance. Gymnastics-specific instruments require psychometric evaluation, including reliability, construct validity, measurement invariance across age and competitive level, and sensitivity to change. Studies should distinguish direct self-efficacy measures from performance-prediction and adapted-confidence proxies, sport confidence, self-confidence, and perceived physical ability, and match the specificity of each measure to the performance outcome.

Future studies should combine standardized judged outcomes with movement-quality, biomechanical, or kinematic indicators where relevant, while controlling for prior performance, training history, competitive level, age, maturation, injury, fatigue, training load, and coach or club effects. Experimental studies of imagery, self-modeling, goal-setting, feedback, or pre-performance routines should use adequate samples, transparent randomization, and outcomes directly relevant to artistic gymnastics.

## 7. Conclusions

This systematic review indicates a possible positive association between task- or apparatus-specific efficacy-related beliefs and artistic gymnastics performance, but the evidence is heterogeneous and of low certainty. In practice, apparatus-specific efficacy ratings should be treated as complementary readiness information and interpreted alongside recent training execution, technical consistency, injury/fatigue status, and coach observations. Stronger conclusions require standardized gymnastics-specific measures, adequately powered longitudinal designs, clearly defined outcomes, and better control of prior performance and other confounders.

## Figures and Tables

**Table 1:** Characteristics of the studies included in the synthesis.

Study	Country, Sample, and Context	Construct/Instrument and Timing	Performance Outcome	Key Findings/Available Statistics	Role in Synthesis
Lee (1982) [25]	Australia; n = 14 girls, 7–12 years (M = 9.7); novice/developmental competitive women’s artistic gymnastics; South Australian Novice Championships.	Gymnasts’ and coach’s predicted apparatus scores; treated as task-/apparatus-specific efficacy-related proxies; non-standardized score-prediction measure; collected 7 days before competition.	Official judged scores on vault, uneven bars, beam, floor, and tumbling, summed as competition performance.	Gymnasts’ estimates were associated with competition score (r = 0.55, *p* = 0.04). Coach’s estimates were stronger (r = 0.79, *p* = 0.002), whereas previous scores were not significant (r = 0.34, *p* = 0.31).	Primary competitive proxy evidence; small sample and non-standardized efficacy-related proxy; supports task-specific expectations but highlights stronger coach/behavioral indicators.
McAuley & Gill (1983) [16]	USA; n = 52 female collegiate gymnasts from seven universities; intercollegiate women’s artistic gymnastics.	Physical Self-Efficacy Scale; task-specific efficacy inventories for vault, beam, bars, and floor; predicted event scores treated as efficacy-related proxies; administered immediately before official timed warm-ups.	Official event scores for vault, beam, bars, and floor.	Task-specific efficacy correlated with event performance: vault r = 0.284 *, beam r = 0.582 ***, bars r = 0.717 ***, floor r = 0.426 **. General PSE was weaker; predicted scores added substantial variance in regressions.	Primary competitive evidence; supports construct specificity and separation among general physical self-efficacy, direct task-specific efficacy, and predicted-score proxies.
Lee (1986) [5]	Australia; n = 16 girls, 8–13 years (M = 10.9); club-level competitive women’s artistic gymnastics; n = 10 competed.	Non-standardized uneven bars expected-score measure used as an apparatus-specific efficacy-related proxy; assessed approximately 2 weeks before competition, with repeated monitoring in six gymnasts.	Competition uneven bars score and number of successfully completed elements.	Among competitors, the expected-score proxy correlated moderately with competition score (r = 0.37) and completed elements (r = 0.49); training performance correlated more strongly with these outcomes (r = 0.63 and r = 0.73).	Primary competitive proxy evidence; the expected-score proxy was informative but weaker than contemporaneous training execution.
Weiss, Wiese, & Klint (1989) [26]	USA; n = 22 boys, 7–18 years; competitive youth men’s artistic gymnastics at state championship level.	Expected event scores used as event-specific efficacy-related proxies; anxiety and worry also assessed; collected approximately 2 h before competition.	Official event and all-around competition scores.	Expected-score proxy correlations varied by apparatus: high bar r = 0.84, pommel horse r = 0.66, floor r = 0.59, parallel bars r = 0.54, still rings r = 0.36, vault r = 0.27; all-around r = 0.71. Regression: beta = 0.63, R^2^ = 0.409.	Primary competitive proxy evidence; strong task-specific findings, but small heterogeneous youth sample and non-independent apparatus effects.
Winfrey & Weeks (1993) [4]	Country not reported; n = 11 intermediate female gymnasts, 8–13 years; gymnastics-specific experimental setting.	Balance beam confidence inventory adapted from the State Sport Confidence Inventory and operationalized for the gymnastics task; treated as an efficacy-related proxy; assessed at pretest and 2-, 4-, and 6-week post-tests.	Expert-rated balance beam routine performance.	No significant between-group differences in the efficacy-related proxy or performance were reported. The self-modeling group’s self-rated versus actual post-test performance correlated at r = 0.92; control group r = 0.02.	Experimental efficacy-related proxy evidence; no clear between-group intervention effect; very small randomized trial interpreted cautiously.
Chase, Magyar, & Drake (2005) [27]	USA; n = 10 female competitive gymnasts, 12–17 years; Levels 7–10; injury history.	Structured qualitative interviews on fear of injury, sources of self-efficacy, and psychological strategies; conducted in the practice context.	No direct judged performance outcome; performance-related fear, readiness, and return-to-skill themes.	Inductive content analysis identified mastery/performance information, communication with significant others, self-awareness, preparation, and comparison as efficacy sources.	Qualitative contextual evidence only; CASP appraised; not included in quantitative association synthesis.
LaForge-MacKenzie & Sullivan (2014) [28]	Canada; n = 47 university students (27 men, 20 women; M age = 20.32, SD = 1.49; range 19–25 years); non-competitive educational gymnastics course.	Moment-to-moment task-specific self-efficacy ratings during a continuous routine; researcher-developed measure; collected immediately before mandatory skills.	Expert-rated body-control scores for routine segments.	Path analysis indicated that self-efficacy did not significantly predict subsequent performance, nor did performance consistently predict subsequent self-efficacy at the next segment.	Educational/non-competitive sensitivity evidence; not combined with competitive synthesis.
Milosis et al. (2018) [29]	Greece; n = 370 physical-education university students (207 male, 163 female); former gymnasts and related athletes excluded.	Skill- and apparatus-specific gymnastics self-efficacy questionnaires; researcher-developed and psychometrically tested; measured at the beginning of the year, end of first semester, and end of academic year.	End-of-year practical gymnastics examination grades.	Self-efficacy correlated with grades across measurements (males r = 0.46/0.58/0.65; females r = 0.56/0.54/0.63). Hierarchical regressions explained approximately R^2^ = 0.44 for males and R^2^ = 0.41 for females.	Educational/non-competitive sensitivity evidence; relevant to gymnastics learning but not directly generalizable to competitive artistic gymnastics.
Aikawa & Takai (2021) [30]	Japan; n = 52 first-division university gymnastics club members (29 men, 23 women; M age = 19.7 +/− 1.1 years); structured training 6 days/week.	Self-efficacy scale (alpha = 0.86) assessed with imagery ability and automatic thoughts; self-efficacy collected 5 days before an internal selection match; imagery/thoughts assessed immediately after competition.	Internal selection-match performance calculated from planned difficulty and actual all-around score.	Self-efficacy showed a weak, non-significant correlation with performance (r = 0.22). Confidence correlated with performance (r = 0.32 *). Goal imagery correlated with both performance (r = 0.43 **) and self-efficacy (r = 0.40 **).	Competitive/club-level evidence; interpreted cautiously as mixed or weaker evidence for a direct self-efficacy-performance association.

Note. Effect estimates and precision estimates are reported only when available in the original studies; no confidence interval was inferred when the source did not report one. PSE = Physical Self-Efficacy Scale. * *p* < 0.05; ** *p* < 0.01; *** *p* < 0.001. Competitive artistic gymnastics studies, experimental evidence, educational/non-competitive studies, and qualitative contextual evidence were distinguished because constructs, samples, timing, and performance outcomes were not directly interchangeable.

**Table 2:** Methodological appraisal of included studies.

Study	Appraisal Tool	Overall Judgment	Main Methodological Considerations
Lee (1982) [25]	NIH Quality Assessment Tool for Observational Cohort and Cross-Sectional Studies, adapted	Poor	Very small sample, non-standardized expected-score efficacy-related proxy, limited control of confounding, incomplete previous-score data, and exploratory regression modelling with limited statistical power.
McAuley & Gill (1983) [16]	NIH Quality Assessment Tool for Observational Cohort and Cross-Sectional Studies, adapted	Fair	Clear competitive sample and official performance outcomes; inclusion of both general and task-specific efficacy measures. Limitations include cross-sectional design and limited control of prior performance, training history, and other confounders.
Lee (1986) [5]	NIH Quality Assessment Tool for Observational Cohort and Cross-Sectional Studies, adapted	Poor	Exploratory small-sample study; only a subsample competed and contributed competition outcomes; single-apparatus focus; limited statistical power and no formal control of confounders.
Weiss, Wiese, & Klint (1989) [26]	NIH Quality Assessment Tool for Observational Cohort and Cross-Sectional Studies, adapted	Fair	Clear competitive context, proximal assessment timing, and official performance outcomes. Main limitations include small sample size, broad age range, and limited adjustment for age, experience, ability level, and other confounders.
Winfrey & Weeks (1993) [4]	Cochrane Risk of Bias 2 tool	Some concerns	Random assignment was reported, but the trial was very small and reporting of the randomization process and implementation details was incomplete. Interpretation is further limited because the adapted confidence measure was an efficacy-related proxy and findings mainly concern within-group associations rather than a clear intervention effect.
Chase, Magyar, & Drake (2005) [27]	CASP Qualitative Studies Checklist	Moderate credibility/contextual only	Qualitative design was appropriate for exploring sources of self-efficacy and fear of injury. Limitations include a small single-club sample, focus on gymnasts with injury histories, limited reflexivity reporting, and absence of quantitative performance outcomes.
LaForge-MacKenzie & Sullivan (2014) [28]	NIH Quality Assessment Tool for Observational Cohort and Cross-Sectional Studies, adapted	Fair	Clear educational design and repeated moment-to-moment assessment. However, participants were non-competitive university students, performance was assessed within an educational routine, and concurrent self-efficacy ratings may have influenced task execution.
Milosis et al. (2018) [29]	NIH Quality Assessment Tool for Observational Cohort and Cross-Sectional Studies, adapted	Fair	Large sample and psychometric testing of skill-specific self-efficacy instruments strengthen the study. Limitations include educational/non-competitive sample, exclusion of experienced gymnasts, use of course grades as performance outcomes, and possible teacher/course-related bias.
Aikawa & Takai (2021) [30]	NIH Quality Assessment Tool for Observational Cohort and Cross-Sectional Studies, adapted	Fair	Competitive university club sample and performance outcome were clearly described. Limitations include modest sample size, self-efficacy not being the main predictor of interest, limited confounder control, and use of an internal selection-match context rather than external official competition.

Note. RoB 2 was used for the randomized controlled trial, the NIH Quality Assessment Tool for non-randomized quantitative studies, and the CASP Qualitative Studies Checklist for the qualitative study. Judgments were used to support cautious interpretation rather than to exclude studies from the synthesis.

## Data Availability

No new primary data were generated. All data extracted for this review are reported in the article and Supplementary Materials.

## Supplementary Materials

The following supporting information can be downloaded at: https://www.mdpi.com/article/10.3390/sports14080343/s1. Supplementary File S1. Completed PRISMA 2020 checklist; Table S1. Recorded search strategies and source details; Table S2. Eligibility decisions for records identified through other methods and two database-derived full-text exclusions listed for transparency; Table S3. Detailed characteristics and statistical findings of included studies; Table S4. Narrative sensitivity checks; Table S5. Methodological appraisal reporting map; Table S6. Narrative confidence-in-evidence summary; Table S7. CASP qualitative appraisal of the qualitative study.

## Abbreviations

The following abbreviations are used in this manuscript:

FIG	Fédération Internationale de Gymnastique
MAP	Multi-Action Plan
NIH	National Institutes of Health
PECOS	Population, Exposure, Comparator, Outcome, and Study Design
PERSIST	PRISMA in Exercise, Rehabilitation, Sport Medicine and Sports Science
PRISMA	Preferred Reporting Items for Systematic Reviews and Meta-Analyses
RoB 2	Risk of Bias 2
