# Supplementary material for: Self-Efficacy and Performance in Artistic Gymnastics: A Systematic Review
Source: Sports (Basel). 2026 Aug 7;14(8):343. doi: 10.3390/sports14080343 (PMC13517257; doi:10.3390/sports14080343)
Supplement: Supplementary file 1 [file sports-14-00343-s001.zip › sports-4398026_PRISMA_2020_Checklist_FM.pdf]

PRISMA 2020 Checklist

Section and Topic	Item #	Checklist item	How it is reported in the manuscript
**TITLE**			
Title	1	Identify the report as a systematic review.	Title: “Self-Efficacy and Performance in Artistic Gymnastics: A Systematic Review” explicitly identifies the report as a systematic review
**ABSTRACT**			
Abstract	2	See the PRISMA 2020 for Abstracts checklist.	Abstract reports the rationale/background, review aim, PRISMA 2020 and PERSIST 2021 reporting framework, PROSPERO registration number, databases searched, time frame, eligibility criteria, number of included studies, main findings, methodological heterogeneity, and conclusions/future research implications.
**INTRODUCTION**			
Rationale	3	Describe the rationale for the review in the context of existing knowledge.	Introduction explains the relevance of self-efficacy for performance in artistic gymnastics, the task- and context-specific nature of the construct, the overlap with confidence-related measures used in gymnastics studies, and the gap created by the absence of a previous systematic synthesis of this evidence.
Objectives	4	Provide an explicit statement of the objective(s) or question(s) the review addresses.	Introduction, final paragraph: the aim is explicitly stated as synthesizing and critically evaluating the existing evidence on the relationship between self-efficacy and performance in artistic gymnastics, with attention to assessment methods, timing and specificity of assessments, and types of performance outcomes considered.
**METHODS**			
Eligibility criteria	5	Specify the inclusion and exclusion criteria for the review and how studies were grouped for the syntheses.	Materials and Methods, Eligibility Criteria and Exclusion Criteria: two population contexts were eligible but synthesized separately—competitive/developmental artistic gymnasts as primary evidence and educational/university cohorts performing explicitly artistic-gymnastics tasks under structured instruction with formal performance assessment as indirect evidence. Non-competitive status alone was not an exclusion criterion. Studies also had to report an eligible task-/performance-specific construct and performance outcome or relationship.
Information sources	6	Specify all databases, registers, websites, organisations, reference lists and other sources searched or consulted to identify studies. Specify the date when each source was last searched or consulted.	Materials and Methods, Sources and Search Strategy, and Table S1: PubMed (National Library of Medicine), Scopus (Elsevier), Web of Science Core Collection (Clarivate), and EBSCOhost (SPORTDiscus and APA PsycInfo) were searched from inception to 18 December 2025. APA PsycInfo is reported using its current name; the registered protocol/search documentation used the historical label 'PsycLIT'. Five designated reviews or meta-analyses and the other-methods candidate set were last checked on 26 July 2026.
Search strategy	7	Present the full search strategies for all databases, registers and websites, including any filters and limits used.	Materials and Methods, Sources and Search Strategy, and Table S1 report the retained search strings, database interfaces, search date, filters, and yields. The combined EBSCOhost yield is reported transparently; separate SPORTDiscus/APA PsycInfo yields and unavailable platform-specific line histories were not reconstructed post hoc.

PRISMA

PRISMA 2020 Checklist

Section and Topic	Item #	Checklist item	How it is reported in the manuscript
Selection process	8	Specify the methods used to decide whether a study met the inclusion criteria of the review, including how many reviewers screened each record and each report retrieved, whether they worked independently, and if applicable, details of automation tools used in the process.	Materials and Methods, Data Collection Process: records were imported into Rayyan; duplicates were automatically detected and removed; two independent reviewers (F.M. and G.D.P.) screened titles/abstracts/keywords and full texts in two stages; disagreements were resolved by discussion and, when needed, by a third reviewer (D.C.). Figure 1 summarizes the selection process.
Data collection process	9	Specify the methods used to collect data from reports, including how many reviewers collected data from each report, whether they worked independently, any processes for obtaining or confirming data from study investigators, and if applicable, details of automation tools used in the process.	Materials and Methods, Data Items: F.M. and G.D.P. extracted and organized data collaboratively in structured tables; extraction was not performed independently in duplicate. Entries and statistics were checked against source reports during preparation of Tables 1 and S3, with discrepancies resolved by discussion. No investigators were contacted and no automation tool was used for extraction.
Data items	10a	List and define all outcomes for which data were sought. Specify whether all results that were compatible with each outcome domain in each study were sought (e.g. for all measures, time points, analyses), and if not, the methods used to decide which results to collect.	Materials and Methods, Eligibility Criteria, Outcome Operationalization and Synthesis Framework, and Data Items: outcomes sought were performance-related measures operationalized as performance efficacy and performance efficiency. Performance efficacy included judged scores, execution scores, apparatus-specific error rates, and discrepancies between predicted and actual performance; performance efficiency included movement-quality or biomechanical indicators when explicitly available.
	10b	List and define all other variables for which data were sought (e.g. participant and intervention characteristics, funding sources). Describe any assumptions made about any missing or unclear information.	Materials and Methods, Data Items, and Table 1: other variables extracted included geographical location, study design, sample characteristics (sample size, age, gender), self-efficacy or confidence-related assessment methods, performance assessment approaches, apparatus when reported, and main findings. Missing or unclear sample size information was noted and considered within risk-of-bias assessment rather than inferred.
Study risk of bias assessment	11	Specify the methods used to assess risk of bias in the included studies, including details of the tool(s) used, how many reviewers assessed each study and whether they worked independently, and if applicable, details of automation tools used in the process.	Materials and Methods, Risk of Bias Assessment: risk of bias was assessed independently by two reviewers (F.M. and G.D.P.). Randomized controlled trials were evaluated using the Cochrane Risk of Bias 2 (RoB 2) tool; observational and cross-sectional studies were assessed using the National Institutes of Health (NIH) Quality Assessment Tool for Observational Cohort and Cross-Sectional Studies. Disagreements were resolved through discussion until consensus.

PRISMA

PRISMA 2020 Checklist

Section and Topic	Item #	Checklist item	How it is reported in the manuscript
Effect measures	12	Specify for each outcome the effect measure(s) (e.g. risk ratio, mean difference) used in the synthesis or presentation of results.	Materials and Methods, Outcome Operationalization and Synthesis Framework, Data Items, Table 1, and Supplementary Table S3: study-level correlations, regression information, or explicit null findings are reported when available in the original studies. No pooled effect measure was calculated because the available estimates did not represent sufficiently comparable construct-outcome relationships. No unreported estimate was calculated or inferred.
Synthesis methods	13a	Describe the processes used to decide which studies were eligible for each synthesis (e.g. tabulating the study intervention characteristics and comparing against the planned groups for each synthesis (item #5)).	Materials and Methods, Eligibility Criteria and Outcome Operationalization and Synthesis Framework; Results, Findings: studies were synthesized according to the predefined eligibility criteria and organized around performance efficacy and performance efficiency dimensions, with interpretation guided by the Multi-Action Plan (MAP) model.
	13b	Describe any methods required to prepare the data for presentation or synthesis, such as handling of missing summary statistics, or data conversions.	Materials and Methods, Data Items and Outcome Operationalization and Synthesis Framework: data preparation consisted of extracting and organizing study characteristics, self-efficacy measures, performance outcomes, apparatus information when reported, and main self-efficacy-performance findings. Missing or unclear information was noted rather than inferred when applicable.
	13c	Describe any methods used to tabulate or visually display results of individual studies and syntheses.	Results, Study Selection, Figure 1, Table 1, Risk of Bias, Figure 2, and Table 2: Figure 1 uses the PRISMA flow structure including reports sought and not retrieved; Table 1 presents study characteristics/findings; Figure 2 presents study-level appraisal judgments and Table 2 gives study-level rationales.
	13d	Describe any methods used to synthesize results and provide a rationale for the choice(s). If meta-analysis was performed, describe the model(s), method(s) to identify the presence and extent of statistical heterogeneity, and software package(s) used.	Materials and Methods, Outcome Operationalization and Synthesis Framework, and Results, Findings: a structured narrative synthesis was conducted rather than meta-analysis because of substantial heterogeneity in self-efficacy assessment, performance outcomes, timing of assessment, and study design. Findings were organized according to theoretically grounded efficacy and efficiency dimensions.
	13e	Describe any methods used to explore possible causes of heterogeneity among study results (e.g. subgroup analysis, meta-regression).	Results, Findings: possible sources of heterogeneity are explored narratively across self-efficacy measurement approaches, predictive role of self-efficacy, competitive levels, context, apparatus, and efficiency-related outcomes. No subgroup analysis or meta-regression was conducted.
	13f	Describe any sensitivity analyses conducted to assess robustness of the synthesized results.	Materials and Methods, Outcome Operationalization and Synthesis Framework; Results, Narrative Synthesis; and Supplementary Table S4: two structured narrative sensitivity checks were conducted—one considering direct self-efficacy measures together with prespecified efficacy-related proxies while maintaining their distinction, and one separating competitive from educational/non-competitive samples.
Reporting bias assessment	14	Describe any methods used to assess risk of bias due to missing results in a synthesis (arising from reporting biases).	Materials and Methods, Sources and Search Strategy: no formal assessment of reporting bias (e.g., publication bias or small-study effects) was conducted because the limited number of included studies and the absence of quantitative synthesis precluded meaningful evaluation.

PRISMA

PRISMA 2020 Checklist

Section and Topic	Item #	Checklist item	How it is reported in the manuscript
Certainty assessment	15	Describe any methods used to assess certainty (or confidence) in the body of evidence for an outcome.	Materials and Methods, Risk of Bias Assessment: confidence in the evidence was summarized narratively from study design, risk of bias, inconsistency, indirectness, imprecision, and reporting limitations. Low/very-low descriptors are explicitly identified as narrative and not formal GRADE ratings.
**RESULTS**			
Study selection	16a	Describe the results of the search and selection process, from the number of records identified in the search to the number of studies included in the review, ideally using a flow diagram.	Results, Study Selection and Figure 1: 884 database records yielded 344 records after 540 duplicates were removed; 305 were excluded at screening; 39 reports were sought and all 39 obtained; 32 were excluded and 7 studies retained. Other methods identified 25 records; all 25 reports were obtained and assessed; 23 were excluded and 2 studies included. The final synthesis contains 9 studies.
	16b	Cite studies that might appear to meet the inclusion criteria, but which were excluded, and explain why they were excluded.	Figure 1 and Supplementary Table S2 identify the screened records and report the reasons for exclusion. Other-method exclusions were 6 review/background, 6 non-artistic discipline, 7 no eligible construct-performance relationship, 1 educational/non-comparable context, and 3 grey/abstract/unverifiable records. Milosis and Siatras (2022) was excluded because it did not report an eligible within-sample self-efficacy-performance association, not because the cohort was educational. Author-year entries in Table S2 are screening-record identifiers rather than citations to supporting evidence.
Study characteristics	17	Cite each included study and present its characteristics.	Results, Characteristics of Included Studies and Table 1: characteristics of the nine included studies are presented, including country, study design, sample, apparatus, self-efficacy measure, performance outcomes, and main findings.
Risk of bias in studies	18	Present assessments of risk of bias for each included study.	Results, Risk of Bias, Figure 2, Table 2, and Tables S5 and S7: study-level judgments and rationales are reported for all included studies. Figure 2 does not claim item-level RoB 2 or NIH reporting. Table S7 provides the item-level CASP appraisal for the qualitative contextual study.
Results of individual studies	19	For all outcomes, present, for each study: (a) summary statistics for each group (where appropriate) and (b) an effect estimate and its precision (e.g. confidence/credible interval), ideally using structured tables or plots.	Results, Characteristics of Included Studies and Findings; Table 1; and Supplementary Table S3: study-level correlations, regression information, or explicit null findings are reported when available in the original reports. Precision estimates are included only when reported by the source study. No unreported estimate was calculated or inferred, and no pooled estimate was produced.
Results of syntheses	20a	For each synthesis, briefly summarise the characteristics and risk of bias among contributing studies.	Results, Findings, together with Results, Risk of Bias, Table 1, and Table 2: the narrative synthesis summarizes contributing studies, study characteristics, risk-of-bias considerations, and the main patterns linking task- and apparatus-specific self-efficacy with performance.

PRISMA

PRISMA 2020 Checklist

Section and Topic	Item #	Checklist item	How it is reported in the manuscript
	20b	Present results of all statistical syntheses conducted. If meta-analysis was done, present for each the summary estimate and its precision (e.g. confidence/credible interval) and measures of statistical heterogeneity. If comparing groups, describe the direction of the effect.	Not applicable. No statistical synthesis or meta-analysis was conducted because of methodological heterogeneity. Results are presented narratively in Results, Findings.
	20c	Present results of all investigations of possible causes of heterogeneity among study results.	Results, Findings, and Supplementary Table S4: possible causes of heterogeneity are examined through structured narrative checks considering explicit self-efficacy/efficacy-expectation measures and sample context (competitive versus educational/non-competitive).
	20d	Present results of all sensitivity analyses conducted to assess the robustness of the synthesized results.	Results, Findings, and Supplementary Table S4: sensitivity analyses consider studies with explicit self-efficacy or efficacy-expectation measures and separately examine competitive versus educational/non-competitive samples.
Reporting biases	21	Present assessments of risk of bias due to missing results (arising from reporting biases) for each synthesis assessed.	Materials and Methods, Sources and Search Strategy; Discussion, Certainty and Interpretation of the Evidence; and Limitations: no formal assessment of publication bias or small-study effects was conducted because the evidence base was small and no quantitative synthesis was performed. The potential for publication or language bias is acknowledged as a review limitation.
Certainty of evidence	22	Present assessments of certainty (or confidence) in the body of evidence for each outcome assessed.	Discussion, Certainty and Interpretation of the Evidence; Limitations; and Table S6: confidence in the evidence is summarized narratively. Low/very-low wording is explicitly described as a narrative descriptor, not a formal GRADE rating.
**DISCUSSION**			
Discussion	23a	Provide a general interpretation of the results in the context of other evidence.	Discussion: the findings are interpreted in relation to the broader literature on self-efficacy, confidence, anxiety regulation, psychobiosocial models of performance, and the specific demands of artistic gymnastics.
	23b	Discuss any limitations of the evidence included in the review.	Discussion, Limitations, and Results, Risk of Bias: limitations of the included evidence are discussed, including heterogeneity of study designs and measures, small number of included studies, predominance of observational designs, limited control of confounders, and scarcity of efficiency-related or biomechanical outcomes.
	23c	Discuss any limitations of the review processes used.	Limitations and Materials and Methods: review-process limitations include English-language/peer-reviewed restrictions, grey-literature exclusion, collaborative rather than independently duplicated extraction, a combined EBSCOhost yield, incomplete platform-specific line histories, and possible missed studies despite supplementary checking.
	23d	Discuss implications of the results for practice, policy, and future research.	Future Directions and Conclusions: implications are discussed for future longitudinal research, validation of gymnastics-specific self-efficacy instruments, integration of efficacy and efficiency performance indicators, use of biomechanical/kinematic measures, and applied monitoring of psychological readiness in artistic gymnastics.

PRISMA

PRISMA 2020 Checklist

Section and Topic	Item #	Checklist item	How it is reported in the manuscript
**OTHER INFORMATION**			
Registration and protocol	24a	Provide registration information for the review, including register name and registration number, or state that the review was not registered.	Materials and Methods: the systematic review was prospectively registered in PROSPERO (CRD420251266838). The registered record also mentioned an exploratory pilot assessment; this manuscript reports only the systematic-review component.
	24b	Indicate where the review protocol can be accessed, or state that a protocol was not prepared.	The protocol is accessible through the PROSPERO record CRD420251266838: https://www.crd.york.ac.uk/PROSPERO/view/CRD420251266838.
	24c	Describe and explain any amendments to information provided at registration or in the protocol.	Materials and Methods: during peer review, supplementary backward reference-list checking and forward citation searching were expanded, two eligible reports were added, and competitive, educational, and qualitative evidence were separated explicitly in synthesis. These refinements did not change the primary review question and were not entered as formal amendments in PROSPERO.
Support	25	Describe sources of financial or non-financial support for the review, and the role of the funders or sponsors in the review.	Funding statement: the research received no external funding. The APC will be partially offset by an MDPI voucher assigned to the corresponding author, with the remaining balance covered by Università degli Studi Niccolò Cusano - Telematica Roma. No funder or sponsor had any role in the conduct or reporting of the review.
Competing interests	26	Declare any competing interests of review authors.	Conflicts of Interest statement: the authors declare no conflicts of interest.
Availability of data, code and other materials	27	Report which of the following are publicly available and where they can be found: template data collection forms; data extracted from included studies; data used for all analyses; analytic code; any other materials used in the review.	Data Availability Statement and Supplementary Materials: extracted study data and statistics are reported in Tables 1 and S3; recorded search strategies and source details in S1; eligibility decisions in S2; sensitivity checks in S4; methodological reporting locations in S5/S7; and narrative confidence in S6. No analytic code or pooled dataset was generated because no meta-analysis was conducted.

From: Page MJ, McKenzie JE, Bossuyt PM, Boutron I, Hoffmann TC, Mulrow CD, et al. The PRISMA 2020 statement: an updated guideline for reporting systematic reviews. BMJ 2021;372:n71. doi: 10.1136/bmj.n71. This work is licensed under CC BY 4.0. To view a copy of this license, visit https://creativecommons.org/licenses/by/4.0/
