# Supplementary material for: Self-Efficacy and Performance in Artistic Gymnastics: A Systematic Review
Source: Sports (Basel). 2026 Aug 7;14(8):343. doi: 10.3390/sports14080343 (PMC13517257; doi:10.3390/sports14080343)
Supplement: Supplementary file 1 [file sports-14-00343-s001.zip › sports-4398026_Supplementary_Materials_FM.pdf]

Supplementary Materials

Self-Efficacy and Performance in Artistic Gymnastics: A Systematic Review

Contents: Table S1. Full search strategies; Table S2. Eligibility decisions for records identified through other methods and two database-derived full-text exclusions listed for transparency; Table S3. Detailed characteristics and statistical findings of included studies; Table S4. Narrative sensitivity checks; Table S5. Methodological appraisal reporting map; Table S6. Narrative confidence-in-evidence summary (not a formal GRADE assessment); Table S7. CASP qualitative appraisal of the qualitative study.

Table S1. Search strategies used for the systematic review

Search strategies are reported as retained in the review files. Database searches covered inception to 18 December 2025. PubMed was searched through the National Library of Medicine interface, Scopus through Elsevier, Web of Science Core Collection through Clarivate, and SPORTDiscus and APA PsycInfo through EBSCOhost. APA PsycInfo is reported using its current database name; the registered protocol and retained search documentation used the historical label 'PsycLIT'. The EBSCOhost result was preserved only as a combined SPORTDiscus/APA PsycInfo export; separate yields and unavailable platform-specific line histories were not reconstructed post hoc. Comparator terms and study design filters were applied during screening rather than in the strings. The designated review sources and other-methods candidate set were last checked on 26 July 2026.

**Database**	**Search strategy**	**Filters / Limits**	**Results**
PubMed (National Library of Medicine; searched 18 December 2025)	("artistic gymnastics" OR "competitive gymnastics" OR gymnast*) AND ("self-efficacy" OR "self efficacy" OR confidence) AND (performance OR "execution score*" OR "execution error*" OR "error rate*" OR "event score*" OR "kinematic stability")	Language: English	31
Scopus (Elsevier; searched 18 December 2025)	TITLE-ABS-KEY(("artistic gymnastics" OR "competitive gymnastics" OR gymnast*) AND ("self-efficacy" OR "self efficacy" OR confidence) AND (performance OR "execution score*" OR "execution error*" OR "error rate*" OR "event score*" OR "kinematic stability"))	Language: English	122
Web of Science Core Collection (Clarivate; searched 18 December 2025)	TS=(("artistic gymnastics" OR "competitive gymnastics" OR gymnast*) AND ("self-efficacy" OR "self efficacy" OR confidence) AND (performance OR "execution score*" OR "execution error*" OR "error rate*" OR "event score*" OR "kinematic stability"))	Language: English	148
EBSCOhost (SPORTDiscus and APA PsycInfo; combined export; searched 18 December 2025)	("artistic gymnastics" OR "competitive gymnastics" OR gymnast*) AND ("self-efficacy" OR "self efficacy" OR confidence) AND (performance OR "execution score*" OR "execution error*" OR "error rate*" OR "event score*" OR "kinematic stability")	Language: English	583

Table S2. Eligibility decisions for records identified through other methods and two database-derived full-text exclusions

Note. The other-methods route comprised 25 records: 2 were included and 23 were excluded, matching Figure 1. Exclusion-category totals are 6 review/background, 6 non-artistic discipline, 7 no eligible construct-performance relationship, 1 educational/non-comparable context, and 3 grey/abstract/unverifiable records. Educational status alone was not an exclusion criterion. Pineda-Espejel et al. (2013) and Gomez-Paloma et al. (2014) were database-derived full-text exclusions and are counted only in the database branch. Identification labels reproduce the contemporaneous review log; unavailable platform-specific queries were not reconstructed. Candidate decisions were last checked on 26 July 2026. Author-year entries in Table S2 identify screened records and document eligibility decisions; they are not citations to evidence used to support the review. Bracketed citation numbers identify sources included in the main manuscript reference list.

Screened record identifier	Source	Population/context	Construct	Outcome	Decision	Reason	Role
McAuley & Gill (1983) [16]	Prior reviews/meta-analyses; backward reference-list checking	Women's intercollegiate artistic gymnastics	General physical self-efficacy, apparatus-specific efficacy, predicted scores	Official event scores	Include	Meets PECOS: artistic gymnastics, competitive collegiate sample, direct/task-specific efficacy measures and performance outcomes	Included in the final synthesis; primary competitive evidence.
Aikawa & Takai (2021) [33]	Forward citation searching	Japanese first-division university gymnastics club	Self-efficacy, imagery, thoughts during competition	Internal selection match performance	Include	Eligible competitive/club sample and performance outcome; self-efficacy was measured but not the main significant predictor.	Included in the final synthesis; mixed/weak direct association interpreted cautiously.
Pineda-Espejel et al. (2013)	Database-search branch	Pan American artistic gymnasts	Competitive State Anxiety Inventory-2 Revised (CSAI-2R) self-confidence and anxiety intensity/direction	No direct judged performance outcome analysed as outcome	Exclude	Athlete calibre is appropriate, but the study does not test a self-efficacy-performance relation and uses a related self-confidence/anxiety construct.	Database-branch full-text exclusion; related construct example only; not counted in the other-methods route.
Gomez-Paloma et al. (2014)	Database-search branch	Female artistic gymnastics; recreational and competitive children	Perceived Physical Ability Scale for Children	No direct judged performance score	Exclude	Related perceived-ability construct and recreational-vs-competitive comparison; no direct performance outcome.	Database-branch full-text exclusion; related perceived-ability evidence only; not counted in the other-methods route.
Grandjean et al. (2002)	Additional search-result screening	Elite Olympic women's artistic gymnastics	No direct self-efficacy or confidence scale; confidence disruption inferred	Olympic all-around scores	Exclude	No direct self-efficacy/confidence measurement. Confidence is inferred from vault-height error natural experiment.	Excluded: no eligible self-efficacy or confidence measure.
Dallas et al. (2019)	Additional open-access screening	Young male artistic gymnasts	CSAI-2 self-confidence	Competition performance / high-low performance comparison	Exclude	Competitive artistic gymnastics and performance are present, but the exposure is self-confidence/anxiety rather than self-efficacy. It is not combined with self-efficacy evidence.	Excluded: broader CSAI-2 self-confidence construct was not eligible as task-specific self-efficacy evidence.
Daroglou (2011)	Additional open-access screening	Greek gymnasts; mixed artistic and rhythmic sample	Self-efficacy and coping skills	Performance grouping	Exclude	Artistic and rhythmic gymnastics data are not clearly separable; population does not meet artistic-gymnastics-only criterion	Excluded: artistic- and rhythmic-gymnastics data were not separable.
Milosis & Siatras (2022)	Additional open-access screening	University students in gymnastics course	Gymnastics-specific self-efficacy	Course performance outcomes	Exclude	The study compared sex groups on self-efficacy and course grades but did not report an eligible within-sample association or predictive analysis linking self-efficacy to performance. Educational status alone was not the exclusion reason, and possible overlap with Milosis et al. (2018) [31] was not assumed.	Excluded after full-text eligibility assessment; not used in the quantitative or contextual synthesis.
De Pero et al. (2013)	Additional open-access screening	Elite TeamGym athletes	Self-efficacy, anxiety, fear of injury	Anxiety/fear-related outcomes; no artistic gymnastics performance synthesis	Exclude	TeamGym is outside artistic gymnastics population and primary outcome is not judged artistic gymnastics performance.	Excluded: TeamGym population.
Santos-Rosa et al. (2022)	Additional open-access screening	Rhythmic gymnastics ensembles	Self-confidence/self-talk related constructs	Rhythmic gymnastics performance	Exclude	Rhythmic gymnastics, not artistic gymnastics.	Excluded: rhythmic-gymnastics population.
De Pero et al. (2022)	Additional open-access screening	Young gymnasts, expertise comparison	Motor creativity and self-efficacy	Motor creativity / expertise differences	Exclude	No direct judged artistic gymnastics performance outcome suitable for the review question.	Excluded: no eligible judged artistic-gymnastics performance outcome.
Frischknecht et al. (2016)	Additional open-access screening	Gymnasts and football players	Sport Confidence Questionnaire validation/adaptation	No performance outcome	Exclude	Instrument validation/adaptation study without self-efficacy-performance or confidence-performance outcome.	Excluded: instrument study without an eligible performance outcome.
Halilaj et al. (2016)	Additional open-access screening	Kosovo artistic gymnasts	CSAI-2R self-confidence/anxiety	No direct performance outcome reported	Exclude	Artistic gymnastics sample, but no direct performance outcome and related confidence/anxiety construct.	Excluded: no eligible performance outcome.

Screened record identifier	Source	Population/context	Construct	Outcome	Decision	Reason	Role
Prastyawan et al. (2023)	Additional open-access screening	Students learning gymnastics/cartwheel	Self-confidence	Learning outcome/self-confidence focus	Exclude	Educational learning context, non-competitive sample, and not compatible with McKay Tier 2-5 criteria.	Excluded: educational/non-competitive learning context.
Parkerson et al. (2015)	Additional grey-literature screening	Youth gymnasts	Training-specific self-efficacy / imagery intervention	Training/self-efficacy focus	Exclude	Thesis/grey literature; not peer-reviewed full article for included corpus.	Excluded: thesis/grey literature.
Williams et al. (2023)	Additional conference-abstract screening	Division I collegiate gymnasts with injuries	Practice/competition self-efficacy post-injury	Injury-related comparison; no performance outcome	Exclude	Conference abstract and injury-only focus; no eligible performance outcome.	Excluded: conference abstract and no eligible performance outcome.
Meenakshi et al. (2023)	Additional search-result screening	Artistic gymnastics players, intervention claim	Self-talk/self-confidence	Performance outcome unclear/not sufficiently verifiable	Exclude	Insufficiently verifiable eligible peer-reviewed evidence and unclear/weakly reported performance outcome for systematic-review inclusion	Excluded: insufficiently verifiable evidence and unclear eligible performance outcome.
White & Bennie (2015)	Additional search-result screening	Youth gymnasts and coaches	Resilience themes including efficacy-related ideas	No direct performance outcome	Exclude	Qualitative resilience study; not a self-efficacy-performance study.	Excluded: non-comparable resilience study without a direct performance outcome.
Tsopani et al. (2011)	Forward citation searching	Young female rhythmic gymnasts	CSAI-2 self-confidence	Rhythmic gymnastics performance	Exclude	Rhythmic gymnastics, not artistic gymnastics.	Excluded: rhythmic-gymnastics population.
Rutkauskaite & Skarbalius (2012)	Forward citation searching	Rhythmic gymnasts	Trait/state confidence measures	Rhythmic gymnastics performance/training variables	Exclude	Rhythmic gymnastics, not artistic gymnastics.	Excluded: rhythmic-gymnastics population.
Hammoudi Nassib et al. (2014), Precompetitive anxiety effect on concentration and performance on elite rhythmic gymnasts	Forward citation searching	Elite rhythmic gymnasts	CSAI-2 self-confidence/anxiety	Rhythmic gymnastics performance	Exclude	Rhythmic gymnastics, not artistic gymnastics	Excluded: rhythmic-gymnastics population.
Lochbaum et al. (2023) [11], Pre-event self-efficacy and sports performance	Major prior reviews/meta-analyses; backward reference-list checking source	Sport-wide systematic review/meta-analysis	Pre-event self-efficacy	Sport performance across sports	Not eligible as a primary study	Review/meta-analysis; used for backward checking and context only	Backward reference-list checking source; not a primary study.
Lochbaum et al. (2022) [10], Revisiting the self-confidence and sport performance relationship	Major prior reviews/meta-analyses; backward reference-list checking source	Sport-wide systematic review/meta-analysis	Self-confidence	Sport performance across sports	Not eligible as a primary study	Review/meta-analysis; used for background and backward reference-list checking only	Backward reference-list checking source; not a primary study.
Jekauc et al. (2025; first published online 2023) [12], The effect of self-confidence on performance in sports	Major prior reviews/meta-analyses; backward reference-list checking source	Sport-wide meta-analysis/narrative review	Self-confidence	Sport performance across sports	Not eligible as a primary study	Review/meta-analysis; not a primary artistic gymnastics study	Backward reference-list checking source; not a primary study.
Craft et al. (2003) [9], CSAI-2 and sport performance	Major prior reviews/meta-analyses; backward reference-list checking source	Sport-wide CSAI-2 meta-analysis	CSAI-2 anxiety and self-confidence	Sport performance across sports	Not eligible as a primary study	Review/meta-analysis; used for background/backward reference-list checking only.	Backward reference-list checking source; not a primary study.
Moritz et al. (2000) [7], Self-efficacy measures and sport performance	Major prior reviews/meta-analyses; backward reference-list checking source	Sport-wide self-efficacy meta-analysis	Self-efficacy	Sport performance across sports	Not eligible as a primary study	Review/meta-analysis; used to check eligible primary studies and support task-specificity rationale	Backward reference-list checking source; not a primary study.
Woodman & Hardy (2003)	Backward reference-list checking / forward citation searching	Sport-wide meta-analysis of competitive sport	State cognitive anxiety and state self-confidence	Sport performance across sports	Not eligible as a primary study	Meta-analysis; not a primary artistic-gymnastics study.	Background record identified during checking; not included as a primary study or as one of the five designated checking sources.

Aggregate primary reasons for excluding the 23 other-method records were review/meta-analysis or background source (n = 6), non-artistic discipline (n = 6), no eligible construct–performance relationship (n = 7), educational/non-comparable context (n = 1), and grey literature, conference abstract, or insufficiently verifiable evidence (n = 3).

Table S3. Detailed characteristics and statistical findings of the included corpus

Study	Country	Sample	Calibre/category	Construct measured	Instrument status	Timing	Performance outcome	Available statistics	Role in synthesis
Lee (1982) [29]	Australia	n = 14 girls; 7-12 yrs (M = 9.7); novice competitive WAG	Competitive/developmental; likely McKay Tier 2	Performance-score expectations; efficacy-related proxy	Non-standardized expected-score efficacy-related proxy; task/apparatus-specific	7 days before competition	Official judged scores across five apparatus, summed	Gymnast estimates vs competition score: r = .55, p = .04; coach estimates r = .79, p = .002; previous scores r = .34, p = .31	Primary competitive proxy synthesis; small sample; non-standardized proxy; limited confounding control.
McAuley & Gill (1983) [16]	USA	n = 52 female collegiate gymnasts from seven universities	Competitive collegiate/intercollegiate; eligible competitive sample	Physical Self-Efficacy Scale; apparatus-specific efficacy inventories; predicted event-score proxies	Validated general PSE scale plus direct task-/apparatus-specific measures; predicted scores treated as efficacy-related proxies	Immediately before official timed warm-ups	Official event scores: vault, beam, bars, floor	Task-specific efficacy correlated with event scores: vault r = .284*, beam r = .582***, bars r = .717***, floor r = .426**. PSE was weaker/less consistent; predicted scores added substantial variance in regressions.	Primary competitive study; supports task specificity and separation of direct measures from predicted-score proxies.
Lee (1986) [5]	Australia	n = 16 girls; 8-13 yrs (M = 10.9); club-level competitive WAG; n = 10 competed	Competitive club; slightly below state level	Uneven-bars expected score; efficacy-related proxy	Non-standardized apparatus-specific expected-score efficacy-related proxy	Training-session assessment approx. 2 weeks before competition; repeated monitoring for six gymnasts	Competition uneven-bars score and number of elements completed	For competitors, expected-score proxy correlated r = .37 with competition score and r = .49 with moves; training performance correlated r = .63 and r = .73 with outcomes; previous scores r = -.21 and r = -.59.	Primary competitive proxy synthesis; expected-score proxy weaker than training performance; small sample.
Weiss, Wiese, & Klint (1989) [30]	USA	n = 22 boys; 7-18 yrs; competitive youth MAG	Competitive youth/state tournament; likely McKay Tier 2-3	Expected event scores used as efficacy-related proxies; worry and anxiety measures	Non-standardized event-specific expected-score efficacy-related proxies	Approx. 2 hours before state championship competition	Official event and all-around scores	Expected-score proxy correlations: high bar r = .84; pommel horse r = .66; floor r = .59; parallel bars r = .54; still rings r = .36; vault r = .27; all-around r = .71. Regression: beta = .63; R2 = .409.	Primary competitive proxy synthesis; strong task-specific associations but small heterogeneous youth sample.
Winfrey & Weeks (1993) [4]	Not reported	n = 11 intermediate female gymnasts; 8-13 yrs	Intermediate competitive academy; competition-oriented	Balance-beam confidence inventory adapted from the State Sport Confidence Inventory; predicted beam score	Adapted task-specific confidence measure treated as an efficacy-related proxy; not independently validated as a gymnastics-specific self-efficacy scale	Pretest, 2-week, 4-week, 6-week post-test	Expert-rated balance beam routine scores	No significant between-group differences in the efficacy-related proxy or performance. Self-modeling group self-rated versus actual post-test performance r = .92; control r = .02.	Experimental efficacy-related proxy evidence; no clear between-group intervention effect; very small randomized trial interpreted cautiously.
Chase, Magyar, & Drake (2005) [27]	USA	n = 10 female competitive gymnasts; 12-17 yrs; Levels 7-10; injury history	Competitive; qualitative contextual evidence	Sources of self-efficacy and fear of injury from structured interviews	Qualitative interview guide; self-efficacy sources; no quantitative efficacy scale	Individual interviews during practice setting	No direct judged performance outcome; performance-related fear/return-to-skill context	Inductive content analysis: performance information, communication with significant others, self-awareness, preparation, and comparison emerged as efficacy sources.	Qualitative contextual evidence only; CASP appraised; not used for quantitative association.
LaForge-MacKenzie & Sullivan (2014) [32]	Canada	n = 47 university students (27 men, 20 women; M age = 20.32, SD = 1.49; range 19–25 years) in an educational gymnastics course	Educational/non-competitive; not McKay Tier 2-5	Concurrent self-efficacy ratings during routine	Researcher-developed task-specific self-efficacy scale for educational task	Five times within one continuous routine	Expert-rated body-control scores	Path analysis: self-efficacy did not significantly predict subsequent performance, nor did performance predict subsequent self-efficacy; previous performance predicted subsequent performance.	Educational/non-competitive sensitivity evidence only; not combined with competitive synthesis.
Milosis et al. (2018) [31]	Greece	n = 370 PE university students (207 male, 163 female); gymnasts excluded	Educational/non-competitive; not McKay Tier 2-5	Skill-specific gymnastics self-efficacy questionnaires by apparatus	Researcher-developed and psychometrically tested skill-/apparatus-specific self-efficacy questionnaires	Beginning of year, end first semester, end academic year	End-of-year practical gymnastics grades	Correlations with grades: males r = .46/.58/.65; females r = .56/.54/.63. Regression explained approx. R2 = .44 males and R2 = .41 females.	Educational/non-competitive sensitivity evidence only; demonstrates task-specific relation in PE training context.
Aikawa & Takai (2021) [33]	Japan	n = 52 university first-division gymnastics club members; 29 men/23 women; M age = 19.7 +/- 1.1 yrs	High-level university club; structured training 6 days/week; internal selection competition	Self-efficacy, imagery ability, automatic thoughts during competition	Self-efficacy scale (alpha = .86) plus sport imagery and automatic-thought measures; self-efficacy not primary exposure	Self-efficacy 5 days before internal selection match; imagery/thoughts immediately after match	Internal selection-match performance calculated from planned difficulty and actual all-around score	Self-efficacy showed a weak, non-significant correlation with performance (r = .22). Confidence correlated with performance (r = .32*). Goal imagery correlated with performance (r = .43**) and self-efficacy (r = .40**); mastery imagery correlated with self-efficacy (r = .55***).	Competitive/club-level study; interpreted cautiously as mixed/null evidence for direct efficacy-performance link.

Note. Effect estimates are reproduced only when reported in the original studies. PSE = Physical Self-Efficacy Scale; WAG = women's artistic gymnastics; MAG = men's artistic gymnastics. * p < .05; ** p < .01; *** p < .001.

Table S4. Narrative sensitivity checks

Sensitivity check	Studies considered	Studies excluded from this check	Interpretive consequence
Direct self-efficacy measures plus prespecified efficacy-related proxies	Lee (1982) [29]; McAuley & Gill (1983) [16]; Lee (1986) [5]; Weiss et al. (1989) [30]; Winfrey & Weeks (1993) [4]; LaForge-MacKenzie & Sullivan (2014) [32]; Milosis et al. (2018) [31]; Aikawa & Takai (2021) [33]	Chase et al. (2005) [27] was retained only for qualitative contextual interpretation. Performance-prediction and adapted-confidence measures were retained as prespecified efficacy-related proxies, not treated as equivalent to direct self-efficacy measures. Broader confidence or perceived-ability results were excluded from this check unless accompanied by an explicit eligible measure.	Positive associations remained in several early competitive studies using direct measures or task-specific proxies and in Milosis et al. [31]; however, the adapted-confidence randomized trial showed no between-group effect, LaForge-MacKenzie and Sullivan [32] found no prospective association, and Aikawa and Takai [33] reported a weak, non-significant direct self-efficacy-performance correlation. The conclusion remained low-certainty, inconsistent, and non-causal.
Competitive artistic gymnastics only / McKay Tier 2-5 compatible	Lee (1982) [29]; McAuley & Gill (1983) [16]; Lee (1986) [5]; Weiss et al. (1989) [30]; Winfrey & Weeks (1993) [4]; Chase (2005) [27]; Aikawa & Takai (2021) [33]	Educational samples (LaForge-MacKenzie & Sullivan (2014) [32]; Milosis et al. (2018) [31]) excluded from primary interpretation	Conclusions become narrower but more defensible. Educational studies are retained only to discuss boundary conditions and measurement issues.

Table S5. Methodological appraisal reporting map

Tool / evidence type	Studies	Reporting location	Interpretation
RoB 2	Winfrey & Weeks (1993) [4]	Overall judgment: Figure 2 and Table 2; study-level rationale: Table 2.	Five RoB 2 domains were considered; no domain-level matrix is presented.
NIH observational/cross-sectional tool	Seven non-randomized quantitative studies	Overall judgments: Figure 2 and Table 2; study-level rationales: Table 2.	Fair/poor judgments support cautious interpretation; no item-level NIH matrix is presented.
CASP qualitative checklist	Chase et al. (2005) [27]	Contextual overall judgment: Figure 2 and Table 2; item-level appraisal: Table S7.	Narrative appraisal; no numerical score was calculated.

Table S6. Narrative confidence-in-evidence summary (not a formal GRADE assessment)

**Evidence domain**	**Studies**	**Summary of evidence**	**Main limitations**	Narrative confidence
Competitive direct self-efficacy measures and prespecified task-/apparatus-specific efficacy-related proxies with judged performance	Lee (1982) [29]; McAuley & Gill (1983) [16]; Lee (1986) [5]; Weiss et al. (1989) [30]; Winfrey & Weeks (1993) [4]; Aikawa & Takai (2021) [33]	Several small studies report positive associations for direct task-specific measures or closely matched efficacy-related proxies; the randomized trial using an adapted-confidence proxy showed no significant between-group effect, and Aikawa and Takai [33] provide mixed evidence.	Serious risk of bias, imprecision, small samples, reliance on non-standardized or adapted proxies in several studies, limited confounding control, historical scoring changes, and inconsistent apparatus coverage.	Limited confidence (low certainty; narrative descriptor)
Educational/non-competitive self-efficacy and performance	LaForge-MacKenzie & Sullivan (2014) [32]; Milosis et al. (2018) [31]	Shows boundary conditions: findings can be null within continuous educational tasks or positive in course-grade contexts.	Non-competitive samples not compatible with McKay Tier 2-5; grading/course effects; limited generalizability to competitive artistic gymnastics.	Very limited for competitive inference; limited within the educational context
Qualitative sources of self-efficacy/fear of injury	Chase et al. (2005) [27]	Contextual evidence identifies mastery/performance information, significant others, self-awareness, preparation, and comparison as sources of efficacy.	Single-club sample, injury-history focus, no performance outcome, qualitative contextual role only.	Limited contextual confidence; not rated for an efficacy–performance association

Note. Confidence descriptors were assigned narratively from study design, risk of bias, inconsistency, indirectness, imprecision, and reporting limitations. They are not formal GRADE ratings.

Table S7. CASP qualitative appraisal for Chase et al. (2005) [27]

CASP item	Judgment	Rationale
1. Clear aims?	Yes	Aims focus on fear of injury, sources of self-efficacy, and psychological strategies in competitive female gymnasts.
2. Qualitative methodology appropriate?	Yes	Interview-based qualitative design is appropriate for exploring experiences and strategies.
3. Research design appropriate?	Yes	Structured open-ended interviews and inductive content analysis align with the exploratory aim.
4. Recruitment strategy appropriate?	Partial	Purposeful sample of information-rich cases, but all participants were from one club and had injury histories
5. Data collected appropriately?	Yes	Individual interviews, standardized guide, pilot interviews, and verbatim transcription were reported.
6. Researcher-participant relationship considered?	Partial	Interviewer role and qualifications are described, but reflexivity is limited
7. Ethical issues considered?	Yes	IRB/permissions/assent-consent procedures are described
8. Data analysis sufficiently rigorous?	Yes	Inductive content analysis, multiple investigators, reliability check, and consensus procedures are described
9. Clear statement of findings?	Yes	Themes and frequencies are clearly reported with supporting quotes
10. Value of research?	Yes, contextual	Useful for interpreting sources of self-efficacy and fear-management strategies, but not evidence of efficacy-performance association.
